# Unmasking the impact of COVID-19 on the mental health of college students: a cross-sectional study

**DOI:** 10.3389/fpsyt.2024.1453323

**Published:** 2024-11-18

**Authors:** Subi Gandhi, Alexandra Jordan, Ryan Glaman, Brendan Morrow

**Affiliations:** ^1^ Department of Medical Lab Sciences, Public Health, and Nutrition Science, Tarleton State University, Stephenville, TX, United States; ^2^ Department of Epidemiology, Human Genetics, & Environmental Science, School of Public Health, The University of Texas Health Science Center, Houston, TX, United States; ^3^ Department of Educational Leadership & Technology, College of Education, Tarleton State University, Stephenville, TX, United States

**Keywords:** COVID-19, depression, anxiety, mental illness, college students, mental health, mental disorder, student health

## Abstract

**Introduction:**

Safeguarding college students’ mental health and well-being poses a challenge for college administrators and clinicians because of the unique circumstances students face and the limited literature on their needs. Prior to the pandemic, depression and anxiety were already on the rise among college students, but the changes associated with it may have exacerbated these issues. Our study aimed to explore factors influencing college students’ mental health and identify common trends in their experiences that could assist organizations and policymakers in the future.

**Methods:**

The study participants (n = 571) were chosen using a convenience sample of undergraduate and graduate students attending a Central Texas university. We employed the Qualtrics survey platform to collect data on multiple demographic variables, behavioral health, and other health outcomes of students in the summer semester of 2021. Our objective in this study was to investigate the various factors that contribute to mental health conditions, particularly anxiety, and depression, independently and jointly, by employing two validated tools - Patient Health Questionnaire 9 (PHQ-9), which measures depression, and General Anxiety Disorder 7 (GAD-7), which measures anxiety.

**Results:**

Both the tools displayed satisfactory internal consistency, with Cronbach’s alpha coefficients (α) of 0.921 for the PHQ-9 (9-item) scale and 0.943 for the GAD-7 (7-item) scale. The prevalence of depression and anxiety among participants was 39.4% and 40.1%, respectively, with 31% of participants overall reporting both conditions. Among the explored factors, age, race, parent/guardian’s level of education, campus residence status, and health insurance status were associated with depression (p < 0.05), whereas gender, age, race, and parent/guardian’s level of education were associated with anxiety (p < 0.05). Academically, several factors related to learning difficulties (i.e., staying motivated to learn, finding a quiet place to learn) were associated with the severity of depression and anxiety severity, demonstrated through composite scores.

**Conclusion:**

Robust longitudinal studies should be carried out to ascertain key influencers that affect students’ mental health, and colleges and universities should create policies and protocols to provide support for students during major catastrophes, such as the COVID-19 pandemic, taking into account these influencing factors.

## 1 Introduction

The likelihood of pandemics has risen due to increased global travel, interactions with urban environments, and environmental exploitation (1–4). Pandemics can lead to significant morbidity, mortality, and economic losses worldwide. Research indicates that many college students suffer from mental health issues, even when indirectly exposed to these disasters (3, 5–7), warranting further examination of this issue.

### 1.1 Determinants of mental health

Mental illness results from a confluence of environmental and biological factors, and the instability of college years may aggravate underlying trauma or make those with genetic predispositions more vulnerable to mental health conditions (8–17). The cumulative occurrence of any psychiatric disorder among college students is 30 to 54% (18–21). More specifically, despite challenges in accurately assessing the mental illness burden in this population (22, 23), prior evidence suggests that the prevalence of depressive disorders among college students ranges from 3.4% to 37.2% (21, 24–27), and the prevalence of generalized anxiety ranges from 6.1% to 32% (21, 26–28).

### 1.2 COVID-19’s impact on college mental health

Mental health has persistently been a major issue for college students across the United States, and this concerning reality predates the onset of the COVID-19 pandemic (29–32). The decline in mental health among students during the pandemic renders this vulnerable population more susceptible to various illnesses and may compromise their immune system over time (33–35). Experiencing mental disorders such as anxiety or depression in early adulthood raises the likelihood of facing these mental health issues years later (35, 36). Furthermore, the comorbidity of mental health disorders such as anxiety and depression has been demonstrated to contribute to cognitive decline and irregular sleep patterns, as well as an increase in self-harm behaviors (37–41). While accurately predicting the next pandemic pathogen is challenging, a critical evaluation of previous efforts to plan for and respond to disease outbreaks may facilitate a more proactive and efficacious response in future scenarios (3, 4). This highlights the imperative to investigate the effects of these comorbidities among college students during and in the post pandemic era.

The stage of emerging adulthood (ages 18–24) for college students is conceptualized as a developmental phase that is correlated with a heightened propensity for risk-taking behaviors and an elevated susceptibility to the onset of mental health disorders (35, 42–44). The COVID-19 pandemic brought forth a significant set of challenges for college students that deeply impacted their mental well-being. Some of the prominent pandemic-associated stressors were related to campus closures, remote learning, basic needs, fear of infection, and uncertainty regarding their academic and career prospects (45–51). A study investigating 1,392 universities in seven countries revealed multiple stressors affecting students and highlighted a growing demand for mental health assistance during the COVID-19 crisis (52).

There is evidence of the severe implications of COVID-19 on students’ mental health in the US and Globally. In the United States, college students exhibited elevated levels of COVID-related anxiety (49.1%), depression (52.3%), increased substance use (24.7%), and recent suicidal ideation (25.5%). This cohort’s rates of suicidal ideation and anxiety were more than twofold compared to those of the overall population (53, 54). Globally, the effects of the pandemic have continued to be felt even in the post-pandemic phase, where issues like depression and anxiety have shown signs of stabilization. However, students have reported an increase in symptoms associated with conditions including post-traumatic stress disorder and suicidal ideation (55).

#### 1.2.1 Online learning and mental health

For college students, one of the most significant transitions during the COVID-19 pandemic was the shift to online learning. Studies have reported elevated levels of stress, anxiety, and depression among students, with the transition to remote learning exacerbating pre-existing mental health concerns (56). Students’ self-efficacy was damaged by the challenges they encountered while studying virtually, and this led to anger, hopelessness, anxiety, and despair (57–61). Moreover, shifts in class schedules, delays in internships, and foregone culminating experiences like practicums and in-person graduation ceremonies also eroded students’ resilience and sense of community, contributing to stress and generalized anxiety (24, 47, 62, 63). While students continued their education, online learning led to feelings of isolation and loneliness. The reduced social interaction also worsened mental health issues due to the absence of in-person support (64). Previous studies have examined the challenges posed by the pandemic on student learning and academic performance (65–67), which could have either initiated or exacerbated college students’ mental health issues (45). A recent meta-analysis that included participants from 19 nations found high levels of anxiety, depression, and stress in remote learning students during COVID-19 was at 58%, 50%, and 71%, respectively. Additionally, the analysis revealed that anxiety and depression rates were notably higher in higher education students compared to those in elementary education (64).

#### 1.2.2 Physical activity and mental health

A growing body of evidence highlights the positive effects of physical activity on mental well-being. Research indicates that engaging in exercise improves mood and boosts self-esteem while reducing stress levels, a known contributor to the exacerbation of mental and physical health issues (68, 69). Other coping mechanisms adopted by the students, in addition to physical activity, is discussed below in section 1.2.8.

The association between physical activity and mental health was particularly salient in the context of the pandemic which seemed to reduce mental distress (70) as well as the probability of infection and mortality (71, 72). For college students, dedicated outdoor activities and exercise were exchanged for time spent online, and the subsequent decrease in physical activity and diminished exposure to sunlight seemed to affect college students’ mental health (65, 73, 74). For instance, a study demonstrated that individuals who engaged in physical activity reported lower levels of anxiety and depression compared to their sedentary counterparts, irrespective of their pre-pandemic activity levels (75). The shutdown of fitness centers and recreational centers, coupled with limitations on outdoor activities, may have further limited students’ physical activities, potentially contributing to mental health issues. Furthermore, sun exposure has been recognized for its potential benefits to mental health through its role in vitamin D synthesis and psychological effects. Research indicates that adequate sun exposure can enhance mood and alleviate symptoms of depression and anxiety, largely attributed to the role of sunlight in stimulating the production of serotonin. This neurotransmitter contributes to feelings of well-being and happiness (73, 74). The prolonged periods of indoor online learning may have influenced students’ emotional states, potentially affecting their psychological well-being.

Prior studies have found a significant inverse relationship between the amount of time spent in sedentary activities and the ability to manage time-related stress effectively (76). Also, engaging in physical activity is positively correlated with positive emotions among university students, even in the face of stressful events, such as the COVID-19 pandemic (77).

#### 1.2.3 Stress and mental health

The Stress-health Theory suggests that stressful occurrences, such as a pandemic, can significantly affect individual physical and mental well-being (51, 78). The COVID-19 pandemic was particularly distressing for college students due to the disruption of their basic needs, interruptions in academic progress, and the loss of social interactions, alongside the heightened risk of contracting the virus and the associated vulnerabilities (51, 79, 80). Additionally, the Health Risk Perception Model posits that stressful events associated with infectious diseases adversely impact mental health through heightened perceived infection risk (51, 81). Stress mediated the relationship between risk perception of COVID-19 among college students, while perceived control mitigated the impact of perceived stress on mental health. Factors such as extended incubation, high transmission, and potential mortality, combined with the lack of treatments, adversely affected risk perceptions and mental health among college students, particularly those with heightened risk perceptions suffering worse mental health outcomes (80, 82, 83).

#### 1.2.4 Social support, isolation and mental health

Social isolation has been identified as a critical factor contributing to mental distress (79, 84–86). The pandemic-induced restrictions such as social distancing and online learning potentially exacerbated feelings of isolation, particularly among college students who typically depend on social interactions for emotional support and academic collaborations (46, 75, 87, 88). Additionally, the absence of face-to-face engagement resulted in heightened feelings of loneliness and disconnection from peers, which are significant predictors of mental health deterioration (46, 89). Concerns about infection were also linked to an increased likelihood of experiencing depressive symptoms (90), that could further restrict their social interaction during isolation. In a study of college students conducted during the pandemic that included nearly 7,000 college students demonstrated that students who self-isolated for some of the time demonstrated a 1.78 times higher likelihood of reporting clinically significant depressive symptoms compared to those who did not self-isolate themselves. Furthermore, students who self-isolated for most or all of the time exhibited 2.12 and 2.27 times higher likelihood of reporting clinically significant depressive symptoms, respectively, compared to their counterparts (91). Research has also indicated that suboptimal indoor environmental quality and limited spatial dimensions during periods of lockdown have adversely influenced the mental health of college students, as demonstrated in a comprehensive study comprising over eight thousand participants (92).

The construct of loneliness, which was exacerbated by the COVID-19 pandemic and related mental health challenges, is substantiated by Cacioppo’s Evolutionary Theory of Loneliness. This theory explains that the lack of perceived social connectedness may culminate, over an extended duration, in significant mental and physical health consequences (93, 94). The outcomes of the serial mediation analysis demonstrated that anxiety and loneliness effectively elucidated the correlation between distress and depression, with 42% of the variance in depression being attributed to these factors (94).

#### 1.2.5 Basic needs and mental health

The protective factors associated with campus living, such as consistent access to nutritious food, housing, and employment, were disrupted, especially during the early phases of the pandemic, which led to the elimination of more than 13% of jobs in higher education, including on-campus student jobs (65, 95). Concurrently, college students encountered significant challenges related to food security and housing stability (96) during this period. Collectively, these stressors may have substantially impacted the mental health of students. A previous study involving approximately 50 thousand college students examined the relationship between food and housing insecurity and mental health outcomes. The research found that students experiencing low or very low food security, as well as housing insecurity, were significantly more likely to report symptoms of moderate to severe major depressive disorder and generalized anxiety disorder compared to those that did not face these challenges (97).

#### 1.2.6 Comorbidities and mental health

In addition to psychiatric illnesses, the COVID-19 outbreak impacted students’ chronic health conditions (98, 99). In a previous study, the prevalence of persistent diseases among adolescents during the COVID-19 pandemic significantly increased, with notable rises in vision disorders by 1.4 times, musculoskeletal system by 2.8 times, nervous system by 7 times, and gynecological conditions by 1.7 times. The insufficient physical activity and elevated stress levels during the pandemic were proposed as contributing factors leading to the increase in these chronic conditions (100). While many studies have focused on the impact of the pandemic on mental health, there is limited research in the United States examining chronic conditions among college students, whether in relation to mental health or independently, especially in the context of the COVID-19 pandemic.

#### 1.2.7 Abuse and mental health

The university campus was a sanctuary for many students, offering educational opportunities, independence, personal growth, and sometimes refuge from difficult home lives. The pandemic forced students to return home, removing these protective factors and exposing them to environments that may have included family conflict, economic hardship, or abuse (65). While students living with parents were less likely to struggle with food insecurity, work, stress, and health (61), they were at higher risk of being exposed to abuse, especially if their family did not respect their sexual or gender identity (101). Following campus closures, one in ten students relocated to environments where they experienced abuse, and one in twenty students relocated to a home in which they did not feel safe and protected (101). The specific types of abuse—verbal, physical, and sexual—must be examined to assess their impact on mental health among college students during the pandemic.

#### 1.2.8 Coping strategies and mental health

Coping strategies with COVID-19 partially mediated the association between certain related lifestyle behaviors and anxiety and depression (102, 103). The coping strategies employed by college students demonstrated significant variability during the pandemic, influenced by numerous challenges presented by the novel online learning environment, social isolation, and uncertainty regarding their future prospects (103). This variability not only existed in the types but also differed by demographic variables concerning gender and ethnicity (103, 104). Some of the key strategies individuals adopted to cope with the stressors of the pandemic were physical activities (discussed above), mindfulness techniques (105–107), creative expressions (108–110), and expressive writing (111).

Research has demonstrated an inverse relationship between college students’ mental health and social connections, suggesting that social relationships may exert a positive influence on mental health symptoms, particularly depressive symptoms, loneliness, and hopelessness (112). However, this effect has been moderated by gender. Studies have indicated that female students were more likely to employ strategies such as avoidance, acceptance, and seeking social support compared to their male counterparts (113–115). A study conducted at a large, ethnically diverse university indicated that the most frequently employed coping mechanisms were acceptance, emotional processing, and social support. Additionally, the study revealed that individuals who utilized avoidance as a coping strategy experienced higher levels of distress compared to those who employed more beneficial approaches, such as humor (103).

Mindfulness-based interventions (MBIs) emerged as a promising approach to mitigate these psychological challenges during the pandemic (116–119). Research indicates that MBIs can effectively improve mental health outcomes by enhancing mindfulness, reducing stress, and alleviating symptoms of anxiety and depression. A study in Taiwan found that a Mindfulness-Based Cognitive Therapy (MBCT) course effectively enhanced college students’ concentration, reduced stress, managed emotions, and altered cellphone usage patterns among college students. Nearly 60% reported improved concentration, 83% noted significant stress reduction, and 86% stated it enhanced their emotional management (120).

#### 1.2.9 Access to mental health services

The COVID-19 pandemic profoundly influenced college students’ ability to access mental health services. Numerous factors played a role in affecting students’ access to these resources. Some students lacked awareness of the costs and the methods to access these services. For others, while telehealth options were available, many still favored in-person consultations (121), which was not always available, especially during lockdowns. Although telehealth emerged as a potential solution for colleges and universities to provide mental health and victim support services to their students, financial constraints rendered these services inaccessible (122). The findings of one study revealed that 62% of surveyed students reported a loss of mental health support, with more severe depressive and anxiety symptoms, suicidal ideation, and sexual minority identity being associated with a higher likelihood of support loss (123).

Considering the intricacies surrounding the mental health of college students and their far-reaching repercussions, it is essential to explore the stressors linked to the pandemic and to create prompt and effective interventions for future major public health emergencies (25, 27, 47). This study aimed to explore the prevalence of depression and anxiety, both individually and concurrently, among university students in Central Texas during the COVID-19 pandemic. Additionally, it sought to identify potential factors that might directly or indirectly influence these mental health issues. To our knowledge, such a comprehensive analysis has not yet been conducted in the existing literature.

Our two main research objectives were:

1. To evaluate the severity of depression and anxiety among college students during the early phases of the pandemic using validated questionnaires.
2. To explore the potential relationships between depression and/or anxiety and other factors, including basic needs (housing and employment), changes in learning modality, challenges in the learning environment, other direct experiences with COVID-19, comorbidities, abuse, and coping strategies.

## 2 Methods

### 2.1 Study location, design, and sample

The study participants were chosen using a convenience sample of undergraduate and graduate students (124). Student participants were invited to participate in the study via an email that was sent to their university-affiliated email addresses, which contained an overview of the study as well as a link to the online Qualtrics survey. Data were also gathered from several campus locations, such as the dining hall, library, and similar spots. However, the online surveys yielded better responses, likely due to reduced foot traffic during the summer months and the ongoing pandemic. Though 828 students participated in the survey, 377 responses were excluded due to missing data, the participants’ age being below 18 years, or non-response to one or both of the mental health inventories (depression and anxiety). The data were collected between June 25, 2021, and July 16, 2021, and a total of 571 students were included in the final analysis.

### 2.2 COVID-19 infection rates and specific restrictions in higher education

The research was carried out at a major 4-year university, primarily consisting of undergraduates, with additional graduate and doctoral students. Students from nearly every county in Texas are represented in the institution’s enrollment, enhancing the study’s generalizability to college students across the state.

In the final two weeks of April 2020, authorities in Texas implemented lockdown measures for businesses deemed non-essential, while the state’s governor declared that educational institutions would remain closed for the rest of the academic year (103). After the spring break of that year, all college classes were taken online in March. During this time, COVID-19 cases were rapidly rising in the state of Texas. When the data collection ended, the cumulative deaths in Texas was 55,625, and the total age-adjusted deaths per 100,000 was 194 (125). On February 25, 2021, the university issued a statement regarding resuming conventional classroom instruction for the fall semester of that year (126).

### 2.3 Survey instrument

To gather insights about the experiences of participants during the COVID-19 pandemic, we developed a survey that featured demographic questions, validated mental health surveys, substance use and abuse surveys, and a section designed to explore participants’ experiences during the initial stages of the pandemic. The online survey was a much practical way to garner data due to (a) the online survey’s ability to access a large number of participants efficiently) and (b) avoiding individual contact with participants during the ongoing COVID-19 pandemic. Although the survey covered many factors, in this paper, we primarily report on the implications of the pandemic on students’ mental health. The first study explaining the barriers to academic and basic needs and vaccination trends has been published elsewhere (65).

The survey’s demographic domain comprised a range of questions related to age, race and ethnicity, gender, first-generation and traditional student statuses (yes/no), degree level (undergraduate, graduate, post-graduate, and other), parental education status, residential status (on/off campus), health insurance status (yes/no), undergraduate classification, and area of study. The second domain contained questions related to the COVID-19 vaccination, the positivity status of students and their families, and comorbidities experienced during COVID-19 illness. Other domains of interest were basic needs, employment, learning challenges, abuse, information streams, coping strategies, and behavioral health.

We primarily employed The Patient Health Questionnaire 9 (PHQ-9) and the General Anxiety Disorder 7 (GAD-7) to explore students’ mental health for depression and anxiety, respectively. Both questionnaires are widely used, reliable, and valid tools for screening the general and student populations (127–130). The PHQ-9 is a nine-item measure of depression in which participants describe the extent to which they have been bothered by various experiences over the previous two weeks (e.g., “Little interest or pleasure in doing things”) using a four-point rating scale (0 = *Not at all*, 1 = *Several days*, 2 = *More than half the days*, 3 = *Nearly every day*). PHQ-9 depression scale scores are computed by summing the values of participants’ responses to all nine questions (i.e., a possible range of 0-27 points). The GAD-7 is a seven-item measure of anxiety in which participants describe the extent to which they have been bothered by various experiences over the previous two weeks (e.g., “Feeling nervous, anxious or on edge”) using the same four-point rating scale as the PHQ-9. GAD-7 anxiety scores are computed by summing the values of participant’s responses to all seven items (i.e., a possible range of 0-21 points).

Within the present study, we examined the reliability of the PHQ-9 and GAD-7 by calculating Cronbach’s alpha (α) coefficients using SPSS Version 27. Both tools had satisfactory internal consistency reliability, with coefficients of α = 0.92 for the PHQ-9 (9-item) scale and α = 0.94 for the GAD-7 (7-item) scale (131). Concerning the validity of the PHQ-9 and GAD-7, prior research has shown these measures to be valid. The PHQ-9 and GAD-7 items were not modified for the purpose of the current study; both measures were utilized in their original formats. This decision was made because alterations to a validated tool can affect their validity (132). Retaining the original structure of these tools enabled us to measure students’ general anxiety and depression symptoms during the COVID-19 pandemic. Other elements of the survey (e.g., COVID diagnosis and/or vaccination information, experience of learning difficulties, etc.) were more specifically tailored to the COVID-19 pandemic context.

To gain a comprehensive understanding of the relationships between variables, we conducted an analysis of depression and anxiety (our primary outcomes of interest) as both continuous and categorical variables. To explore the categorical relationships of our outcomes of interest with other factors, the PHQ-9 scores were selected to represent mild, moderate, moderately severe, and severe depression at 5, 10, 15, and 20, respectively (127, 128, 133). Similarly, the GAD-7 scores were set to indicate mild, moderate, and severe anxiety at 5, 10, and 15 (128, 134). Based on recent recommendations for screening in non-psychiatric populations, we used the cut point of 10 for PHQ-9 (127, 133) to indicate whether participants were classified as having depression (i.e., a yes/no binary) and a cut point of 8 for GAD-7 (135, 136) to indicate whether participants were classified as having anxiety (i.e., a yes/no binary). Participants who were classified as having both depression and anxiety based on those cut points were further classified as having both conditions (i.e., once again using a yes/no binary for having both anxiety and depression). We also developed a composite score denoting participants’ total PHQ-9 and GAD-7 scores to evaluate the effect of our outcome variables as continuous variables. This not only allowed us to view medians and averages among subgroups but also analyze the severity more thoroughly associated with various factors.

#### 2.3.1 Data management and analysis

Data analysis was conducted using SPSS version 27 (137). Missing data were handled using pairwise deletion to retain as much available data as possible. Although missing data can adversely affect the accuracy of inferences made in inferential statistical tests (138), our fairly large sample size (n = 571) may have circumvented this issue. The deidentified data were imported from Qualtrics into SPSS for cleaning and final analysis.

We conducted a variety of nonparametric analyses to examine the relationships among study variables such as demographic characteristics, anxiety and depression, learning difficulties, and COVID-19-related variables. Specifically, chi-square tests were used to examine relationships among categorical variables. Mann-Whitney U tests were used to examine group differences in study variables when comparing two groups. Kruskal-Wallis tests were used to examine group differences when there were three or more groups. This study used Nonparametric analyses because many of the study variables were non-normally distributed, rendering more typical parametric tests (e.g., t-tests, ANOVA, etc.) inappropriate for use (139). Osborne (2013) (140) suggested that skewness and/or kurtosis statistics in excess of ±1.0 indicate a non-normally distributed variable, and depression and anxiety scores exceeded these thresholds for several groups (for example, Asian participants’ depression composite scores had skewness = 0.32 and kurtosis = -1.85). Therefore, nonparametric tests were more appropriate for the current study.

## 3 Results

The relationships of primary outcomes of interest (depression and anxiety) as binary variables (yes/no) with other demographic characteristics are shown in **Table 1**. In the total sample, the majority of participants were female (72%) and between 18-23 years old (62.1%). Additionally, most were either Caucasian (62.2%), traditional (69.4%), undergraduate students (71.6%) or lived off-campus (68.1%). Nearly 45% of the participants were first-generation students. Other variables explored included undergraduate classification, parental education, and program affiliation.

**Table 1:** Demographic and other characteristics breakdown of participants by depression/anxiety case status: independent and combined analysis (N = 571).

Variable	Sample (n = 571)	^Depression Case (n = 225)			^^Anxiety Case (n = 229)			#Anxiety and Depression Case (n = 177)			Not a Case (n = 294)		
	N (%)	N (%)	V	p	N (%)	V	p	N (%)	V	p	N (%)	V	p
**Gender**			0.08	0.15		0.10	0.05		0.06	0.32		0.12	0.01*
Male	145 (25.4%)	51 (22.7%)			51 (22.3%)			41 (23.2%)			84 (28.6%)		
Female	411 (72.0%)	165 (73.3%)			168 (73.4%)			129 (72.9%)			207 (70.4%)		
Other	15 (2.3%)	9 (4.0%)			10 (4.4%)			7 (4.0%)			3 (1.0%)		
Missing	0 (0.0%)	0 (0.0%)			0 (0.0%)			0 (0.0%)			0 (0.0%)		
**Age**			0.14	0.08		0.19	< 0.01*		0.19	< 0.01*		0.14	0.11
18	42 (7.4%)	16 (7.1%)			11 (4.8%)			9 (5.0%)			24 (8.2%)		
19	77 (13.5%)	26 (11.6%)			33 (14.4%)			23 (13.0%)			41 (13.9%)		
20	81 (14.2%)	32 (14.2%)			36 (15.7%)			27 (15.3%)			40 (13.6%)		
21	74 (13.0%)	34 (15.1%)			40 (17.5%)			33 (18.6%)			33 (11.2%)		
22-23	81 (14.2%)	37 (16.4%)			35 (15.3%)			31 (17.5%)			40 (13.6%)		
24-31	84 (14.7%)	41 (18.2%)			38 (16.6%)			29 (16.4%)			34 (11.6%)		
32+	94 (16.5%)	27 (12.0%)			25 (10.9%)			16 (9.0%)			58 (19.7%)		
Missing	38 (6.7%)	12 (5.3%)			11 (4.8%)			9 (5.1%)			24 (8.2%)		
**Race**			0.17	0.01*		0.15	0.04*		0.17	0.01*		0.15	0.04*
Caucasian	355 (62.2%)	123 (54.7%)			138 (60.3%)			100 (56.5%)			194 (66.0%)		
African American	74 (13.0%)	43 (19.1%)			40 (17.5%)			35 (19.8%)			27 (9.2%)		
Hispanic	103 (18.0%)	45 (20.0%)			42 (18.3%)			35 (19.8%)			51 (17.3%)		
Asian	7 (1.2%)	3 (1.3%)			3 (1.3%)			3 (1.7%)			4 (1.4%)		
American Indian	5 (0.9%)	3 (1.3%)			2 (0.9%)			1 (0.6%)			1 (0.3%)		
East Indian	2 (0.4%)	0 (0.0%)			1 (0.4%)			0 (0.0%)			1 (0.3%)		
Other	24 (4.2%)	8 (3.6%)			3 (1.3%)			3 (1.7%)			16 (5.4%)		
Missing	1 (0.2%)	0 (0.0%)			0 (0.0%)			0 (0.0%)			0 (0.0%)		
**Parent/Guardian’s Education Level**			0.18	<.01*		0.14	0.11		0.16	0.03*		0.156	0.03*
Some high school	65 (11.4%)	40 (17.8%)			38 (16.6%)			33 (18.6%)			20 (6.8%)		
High school	96 (16.8%)	35 (15.6%)			38 (16.6%)			27 (15.3%)			50 (17.0%)		
Some college	119 (20.8%)	44 (19.6%)			43 (18.8%)			36 (20.3%)			68 (23.1%)		
Associate’s degree	52 (9.1%)	23 (10.2%)			19 (8.3%)			16 (9.0%)			26 (8.8%)		
Bachelor’s degree	154 (27.0%)	58 (25.8%)			59 (25.8%)			45 (25.4%)			82 (27.9%)		
Master’s degree	67 (11.7%)	20 (8.9%)			27 (11.8%)			16 (9.0%)			26 (8.8%)		
Doctorate degree	11 (1.9%)	3 (1.3%)			4 (1.8%)			3 (1.7%)			7 (2.4%)		
Missing	7 (1.2%)	2 (0.9%)			1 (0.4%)			1 (0.6%)			15 (5.1%)		
**Non-Traditional Students**			0.01	0.72		0.09	0.04*		0.08	0.05		0.025	0.55
Yes	116 (20.3%)	64 (28.4%)			56 (24.5%)			42 (23.7%)			88 (29.9%)		
No	396 (69.4%)	159 (70.7%)			171 (74.7%)			133 (75.1%)			199 (67.7%)		
Missing	59 (10.3%)	2 (0.9%)			2 (0.9%)			2 (1.1%)			7 (2.4%)		
**First Generation Students**			0.03	0.41		0.05	0.19		0.06	0.17		0.035	0.40
Yes	254 (44.5%)	95 (42.2%)			94 (41.1%)			71 (40.1%)			136 (46.3%)		
No	316 (55.3%)	129 (57.3%)			134 (58.5%)			105 (59.2%)			158 (53.7%)		
Missing	1 (0.2%)	1 (0.4%)			1 (0.4%)			1 (0.6%)			0 (0.0%)		
**Undergraduate Classification**			0.04	0.83		0.09	0.3		0.08	0.37		0.06	0.70
Freshman	45 (7.9%)	17 (7.6%)			13 (5.7%)			11 (6.2%)			26 (8.8%)		
Sophomore	91 (15.9%)	34 (15.1%)			41 (17.9%)			27 (15.3%)			43 (14.6%)		
Junior	143 (25.0%)	60 (26.7%)			62 (27.1%)			50 (28.3%)			71 (24.1%)		
Senior	172 (30.1%)	73 (32.4%)			74 (32.3%)			63 (35.6%)			88 (29.9%)		
Missing	120 (21.0%)	41 (18.2%)			39 (17.0%)			26 (14.7%)			66 (22.4%)		
**Degree Level**			0.07	0.45		0.12	0.05		0.13	0.02*		0.065	0.49
Undergraduate	409 (71.3%)	170 (75.6%)			177 (77.3%)			142 (80.2%)			204 (69.4%)		
Graduate	144 (25.2%)	49 (21.8%)			46 (20.1%)			32 (18.1%)			81 (27.6%)		
Postgraduate	14 (2.5%)	5 (2.2%)			6 (2.2%)			3 (1.7%)			6 (2.0%)		
Other	3 (0.5%)	1 (0.4%)			0 (0.0%)			0 (0.0%)			2 (0.7%)		
Missing	1 (0.2%)	0 (0.0%)			0 (0.0%)			0 (0.0%)			1 (0.3%)		
**College**			0.11	0.23		0.11	0.20		0.14	0.05*		0.112	0.21
Agricultural and Environmental Sciences	93 (16.3%)	39 (17.3%)			40 (17.5%)			29 (16.4%)			43 (14.6%)		
Business	106 (18.6%)	41 (18.2%)			43 (18.8%)			32 (18.1%)			54 (18.4%)		
Education	92 (16.1%)	44 (19.6%)			44 (19.2%)			35 (19.8%)			39 (13.3%)		
Health Sciences and Human Services	123 (21.4%)	38 (16.9%)			38 (16.6%)			25 (14.1%)			72 (24.5%)		
Liberal and Fine Arts	79 (13.8%)	33 (14.7%)			34 (14.9%)			31 (17.5%)			43 (14.6%)		
Science and Technology	77 (13.5%)	30 (13.3%)			30 (13.1%)			25 (14.1%)			42 (14.3%)		
Missing	1 (0.2%)	0 (0.0%)			0 (0.0%)			0 (0.0%)			1 (0.3%)		
**Campus Residence**			0.07	0.10		0.08	0.05		0.07	0.11		0.09	0.04*
On-campus	181 (31.7%)	80 (35.6%)			83 (36.2%)			64 (36.2%)			82 (27.9%)		
Off-campus	389 (68.1%)	144 (64.0%)			145 (63.3%)			112 (63.3%)			212 (72.1%)		
Missing	1 (0.2%)	1 (0.4%)			1 (0.4%)			1 (0.6%)			0 (0.0%)		
**Health Insurance**			0.07	0.10		0.03	0.53		0.05	0.2		0.05	0.29
Yes	453 (79.3%)	171 (76.0%)			179 (78.2%)			135 (76.3%)			238 (81.0%)		
No	117 (20.5%)	54 (24.0%)			50 (21.8%)			42 (23.7%)			55 (18.7%)		
Missing	1 (0.2%)	0 (0.0%)			0 (0.0%)			0 (0.0%)			1 (0.3%)		

^PHQ 9 >=10 (with or without anxiety); ^^GAD-7>=8 (with or without depression); # PHQ-9 <10 & GAD-7 >= 8; ^, ^^, # All measured as binary variables (yes/no).

*Statistically significant at p < 0.05.

### 3.1 Prevalence and severity of depression and anxiety

The results of the bivariate analyses of the outcome (defined as cases meeting the clinical threshold for either illness, as measured by the PHQ-9 or GAD-7) and other demographic variables are also depicted in **Table 1**. The prevalence of depression and anxiety was comparable, with 225 (39.4%) meeting or exceeding the clinical cutoff for depression (“depression cases”) (PHQ-9 >= 10) and 229 (40.1%) meeting or exceeding the clinical cutoff for anxiety (“anxiety cases”) (GAD-7 >= 8). Additionally, 177 (31%) met or exceeded both clinical cutoffs (“comorbid cases”).

**Figure 1:**
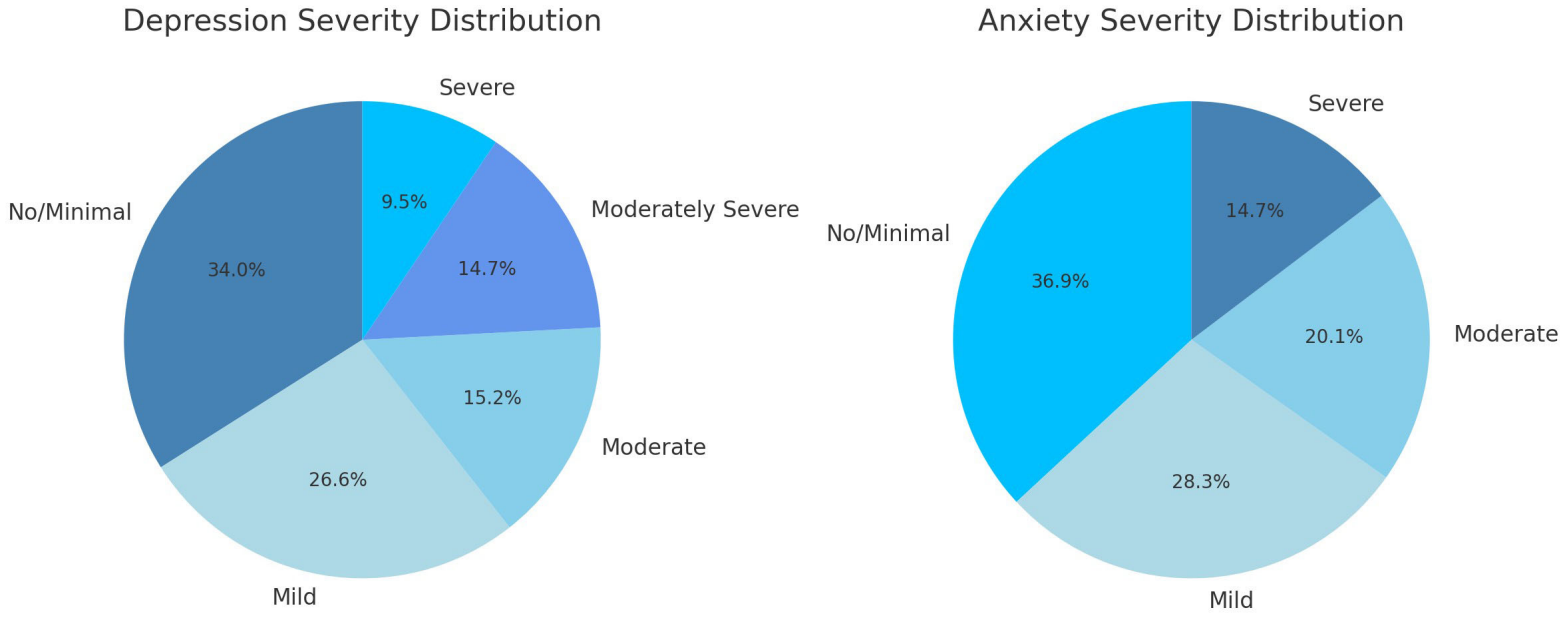
Distribution of depression and anxiety cases by severity (n=571).

**Supplementary Table S1** and **Figure 1** examine categorical severity (none/mild to severe) for both depression and anxiety, and **Table 2** displays these data on a continuous scale as a composite. The mean totals for both the depression and anxiety were just shy of the clinical cutoff ( $\bar{\text{x}}$ = 8.9 and 7.6, respectively), though the median scores were well within subclinical range (M = 8 and 6).

**Table 2:** Demographic and other characteristics distribution of participants according to depression or anxiety severity (N = 571).

Variable	N	Composite Depression Score				Composite Anxiety Score			
		$\bar{\text{x}}$	M	KW/MW	p	$\bar{\text{x}}$	M	KW/MW	p
**Gender**				3.67	0.16			7.96	0.02*
Male	145 (25.4%)	8.49	7.00			6.60	5.00		
Female	411 (72.0%)	8.95	8.00			7.80	7.00		
Other	15 (2.6%)	11.53	13.00			10.20	10.00		
**Age**				19.21	< 0.01*			20.34	< 0.01*
18	42 (7.9%)	7.79	8.00			5.74	5.00		
19	77 (14.4%)	8.73	7.00			7.62	7.00		
20	81 (15.2%)	9.17	8.00			8.04	7.00		
21	74 (13.9%)	10.93	9.00			9.15	8.00		
22-23	81 (15.2%)	9.79	9.00			8.33	7.00		
24-31	84 (15.8%)	9.75	9.00			8.45	7.00		
32+	94 (17.6%)	6.49	5.00			5.82	5.00		
**Race**				21.30	< 0.01*			13.06	0.04*
Caucasian	355 (62.3%)	8.13	7.00			7.19	6.00		
African American	74 (13.0%)	11.05	11.00			9.12	9.00		
Hispanic	103 (18.1%)	10.20	8.00			8.10	6.00		
Asian	7 (1.2%)	12.29	9.00			9.71	5.00		
American Indian	5 (0.9%)	11.80	14.00			8.20	5.00		
East Indian	2 (0.4%)	1.00	1.00			6.00	6.00		
Other	24 (4.2%)	6.96	5.00			4.96	4.00		
**Parent/Guardian’s Education Level**				17.61	< 0.01*			13.94	0.03*
Some high school	65 (11.5%)	12.39	12.00			9.70	10.00		
High school	96 (17.0%)	8.69	7.00			7.29	5.00		
Some college	119 (21.1%)	8.49	7.00			7.40	6.00		
Associate’s degree	53 (9.4%)	9.35	8.50			7.21	5.00		
Bachelor’s degree	154 (27.3%)	8.17	8.00			7.03	6.00		
Master’s degree	67 (11.9%)	8.30	6.00			7.54	6.00		
Doctorate degree	11 (1.9%)	6.73	4.00			6.55	4.00		
**Non-Traditional Students**				35302.00	0.17			35,736.50	0.10
Yes	116 (22.7%)	8.31	7.00			7.05	6.00		
No	396 (77.3%)	9.17	8.00			7.79	7.00		
**First Generation Students**				42007.50	0.34			41,919.50	0.36
Yes	254 (49.6%)	8.53	7.00			7.20	6.00		
No	316 (61.7%)	9.19	8.00			7.81	7.00		
**Undergraduate Classification**				2.44	0.75			1.23	0.49
Freshman	45 (10.0%)	8.02	6.00			6.36	5.00		
Sophomore	91 (20.2%)	9.20	9.00			7.76	7.00		
Junior	143 (31.7%)	9.18	7.00			7.71	7.00		
Senior	172 (38.1%)	9.49	8.00			8.10	7.00		
**Degree Level**				4.00	0.26			4.06	0.26
Undergraduate	409 (71.8%)	9.30	8.00			7.86	7.00		
Graduate	144 (25.3%)	8.01	7.00			6.83	5.00		
Postgraduate	14 (2.5%)	7.29	7.00			6.86	5.50		
Other	3 (0.5%)	5.33	6.00			3.00	4.00		
**College**				5.34	0.38			7.64	0.18
Agricultural and Environmental Sciences	93 (16.3%)	9.44	9.00			8.19	7.00		
Business	106 (18.6%)	8.64	7.00			7.10	6.00		
Education	92 (16.1%)	9.82	9.00			8.82	7.00		
Health Sciences and Human Services	123 (21.6%)	7.80	6.00			6.56	5.00		
Liberal and Fine Arts	79 (13.9%)	9.16	7.00			7.71	6.00		
Science and Technology	77 (13.5%)	9.09	9.00			7.40	7.00		
**Campus Residence**				31599.50	0.048*			32,330.00	0.12
On-campus	181 (31.7%)	9.69	9.00			8.05	7.00		
Off-campus	389 (68.2%)	8.50	7.00			7.31	6.00		
**Health Insurance**				29793.50	0.04*			27,729.50	0.44
Yes	453 (79.5%)	8.62	7.00			7.44	6.00		
No	117 (20.5%)	9.97	9.00			8.00	6.00		

*Statistically significant at p < 0.05.

#### 3.1.1 Demographic influences on depression

Depression analyzed on a case/non-case basis revealed several patterns (see **Table 1**). Students with depression had an average composite score of 16.4 and were predominantly female (73.3%), Caucasian (54.7%), undergraduate (75.6%), or traditional students (70.7%). They often were not the first in their family to attend college (57.3%), and most also lived off campus (64%) or had health insurance (76%). Among all the demographic factors studied, only race and parental education were associated with depression case status (p < 0.05).

We were also interested in the influence of the demographic factors on participants’ composite depression score (also referred to here as “severity”). As demonstrated in **Table 2**, the greatest variations in scores were among different races, though the highest scores were among Asians ( $\bar{\text{x}}$ = 12.3, M = 9), African Americans ( $\bar{\text{x}}$ = 11.1, M = 11), and Hispanics ( $\bar{\text{x}}$ = 10.2, M = 8). Although Caucasians represented the largest group, Hispanics and African Americans experienced the highest rates of depression, anxiety, and comorbid depression and anxiety. Specifically, among Hispanics, the prevalence of depression was 43.7%, anxiety was 57.3%, and comorbid depression and anxiety was 34%. For African Americans, the rates were even higher, with depression at 57.3%, anxiety at 53.3%, and comorbid depression and anxiety at 46.7%. Similarly, high prevalences were found among Asians, but the small sample size limited our ability to make inferences regarding this (n = 7).

Other patterns were found among those with differing parental educational attainment. On average, participants whose parents had only completed some high school had the highest depression score ( $\bar{\text{x}}$ = 12.4), followed by those with parents who had completed an Associate degree ( $\bar{\text{x}}$ = 9.4), and those with parents who had attended some college ( $\bar{\text{x}}$ = 8.5). The Kruskal-Wallis and Mann-Whitney U nonparametric tests revealed that age, race, parental educational attainment, campus residency status (on/off campus), and health insurance coverage were all associated with depression (p < 0.05).

#### 3.1.2 Demographic influences on anxiety

Similar to depression, anxiety analyzed on a case/non-case demonstrated similar patterns (see **Table 1**). Among those with anxiety, the average score was 14.1. Students with anxiety were predominantly female (73.4%), Caucasian (60.3%), undergraduate (77.3%), and traditional (74.7%) students who were not the first in their family to attend college (58.5%). Similarly, 63.3% lived off-campus, and 78.2% had health insurance. Among all the demographic factors examined, only age, race, and being a traditional student demonstrated associations with anxiety (p < 0.05).

Similar to our depression analysis, we also examined anxiety severity as a composite variable (see **Table 2**). Once again, differences were noted among races, with Asians having the highest total score ( $\bar{\text{x}}$ = 9.7, M = 5). Furthermore, the distribution of anxiety scores based upon parental educational attainment was significant but different than that of depression scores. Participants whose parents completed the terminal degree had the lowest score ( $\bar{\text{x}}$ = 7.2, M = 5), and participants whose parents completed some high school had the highest score ( $\bar{\text{x}}$ = 9.7, M = 10). The Kruskal-Wallis and Mann-Whitney U nonparametric tests revealed that gender, age, race, and parental educational attainment were associated with depression (p < 0.05).

#### 3.1.3 Demographic characteristics of those with both anxiety and depression

The data presented in **Table 1** depicts the demographic features of comorbid (anxiety and depression) cases (n =177) assessed on a case/non-case basis. The majority of those with comorbid depression and anxiety were female (72.9%), Caucasian (56.5%), undergraduate (80.2%), traditional students (75.1%), and were not the first person in their family to go to college (59.3%). They also predominantly lived off-campus (63.3%) or had health insurance (76.3%). Initial examination using chi-square tests indicates that age and race influence comorbid case status, with 20–31-year-olds and Black students being slightly overrepresented. However, upon analyzing comorbid cases as a group using Kruskal-Wallis testing, none of the demographic variables seemed to influence the severity of either illness (**Supplementary Table S2**).

Participants suffering from both depression and anxiety had much more severe expressions of either illness. On average, comorbid cases scored higher on the PHQ-9 n ( $\bar{\text{x}}$ = 14.9, M = 16) and GAD-7 ( $\bar{\text{x}}$ = 17.3, M = 13.7; data not shown). Although some degree of variation in composite scores was observed across all the categories of demographic factors, no significant associations were observed between the explored factors and the two mental health conditions (p > 0.05) (**Supplementary Table S2**).

### 3.2 Academics

The academic difficulties students faced, along with corresponding effects on composite depression and anxiety scores, are presented in **Table 3**. The most frequently reported issues were maintaining motivation to learn (66.2%), collaborating with peers for group projects and assignments (49.2%), communicating with instructors (44.7%), and finding a suitable place to study (41%). Most variables were linked to an increase in the severity of depression, anxiety or both (p < 0.05).

**Table 3:** Impact of academic barriers, family circumstances, wellness, and COVID-19 related factors on depression and anxiety severity (N = 571).

	N	Composite PHQ-9 (Depression) Score				Composite GAD-7 (Anxiety) Score			
		$\bar{\text{x}}$	M	MW	p	$\bar{\text{x}}$	M	MW	p
**Overall**				29,285.50	< 0.01*			28,100.50	< 0.01*
Experienced at least one learning difficulty	490 (85.8%)	9.65	9.00			8.12	7.00		
Did not	81 (14.2%)	4.37	2.00			4.10	2.00		
**Type of learning difficulty**									
Staying motivated to learn				51,800.50	< 0.01*			49,547.50	< 0.01*
Yes	378 (66.2%)	10.57	9.00			8.75	7.00		
No	193 (33.8%)	5.62	4.00			5.21	3.00		
**Interacting with peers for group projects and assignments**				50,290.50	< 0.01*			48,223.00	< 0.01*
Yes	281 (49.2%)	10.26	9.00			8.38	7.00		
No	290 (50.8%)	7.58	6.00			6.75	5.00		
**Having difficulty communicating with instructors**				28,731.00	< 0.01*			29,255.50	< 0.01*
Yes	255 (44.7%)	10.8	10.00			9.14	8.00		
No	315 (55.3%)	7.37	6.00			6.27	5.00		
**Finding a quiet place to learn**				47,684.00	< 0.01*			48,744.00	< 0.01*
Yes	234 (41.0%)	10.24	9.00			8.98	7.00		
No	337 (59.0%)	7.96	7.00			6.56	5.00		
**Having high-speed internet access/connection**				43,713.50	< 0.01*				
Yes	201 (35.2%)	10.17	9.00			8.58	7.00	42,945.50	< 0.01*
No	370 (64.8%)	8.21	7.00			7.00	6.00		
**Being too emotionally disturbed to focus on academics**				50,260.50	< 0.01*			49,522.50	< 0.01*
Yes	200 (35.0%)	11.6	11.00			9.76	8.00		
No	371 (65.0%)	7.44	5.00			6.36	5.00		
**Having difficulty balancing work with academics**				42,326.50	< 0.01*			40,393.00	< 0.01*
Yes	200 (35.0%)	10.53	9.00			8.67	7.00		
No	371 (65.0%)	8.15	7.00			7.04	6.00		
**Having difficulty balancing household responsibilities with academics**				44,586.00	< 0.01*			43,239.50	< 0.01*
Yes	190 (33.2%)	10.72	9.00			8.98	7.00		
No	381 (66.7%)	8.00	6.00			6.84	6.00		
**Having a good quality microphone or camera on my computer**				29,913.00	< 0.01*			30,086.00	< 0.01*
Yes	111 (19.4%)	10.30	9.00			8.93	8.00		
No	460 (80.6%)	8.56	7.00			7.22	6.00		
**Not knowing where to get help for academic success**				28,171.00	< 0.01*			26,471.50	< 0.01*
Yes	94 (16.5%)	11.55	12.00			9.10	8.00		
No	477 (83.5%)	8.38	7.00			7.25	6.00		
**Having to take care of relatives or family members during the pandemic (but not related to COVID-19)**				27,971.50	0.01*			27,932.50	0.01*
Yes	103 (18.0%)	10.42	9.00			8.99	7.00		
No	468 (82.0%)	8.56	7.00			7.24	6.00		
**Inability to find student support services when needed**				24,498.50	< 0.01*			23,660.50	< 0.01*
Yes	75 (13.1%)	12.24	14.00			9.96	10.00		
No	496 (86.9%)	8.39	7.00			7.19	6.00		
**Being too physically unwell to focus on academics (but not related to COVID-19)**				26,358.00	< 0.01*			25,572.00	< 0.01*
Yes	80 (14.0%)	12.34	12.50			10.34	9.50		
No	491 (86.0%)	8.34	7.00			7.10	6.00		
**Having been diagnosed with COVID-19 (self)**				16,382.50	0.90			16,971.00	0.55
Yes	64 (11.2%)	8.69	8.00			7.78	6.50		
No	507 (88.8%)	8.93	8.00			7.52	6.00		
**Having to take care of relative or family member that had COVID-19**				16,981.00	0.02*			17,844.50	< 0.01*
Yes	55 (9.6%)	10.89	9.00			10.29	9.00		
No	516 (90.4%)	8.69	8.00			7.26	6.00		
**Having to babysit my siblings**				7,914.00	0.95			7,525.50	0.70
Yes	29 (5.1%)	8.83	8.00			7.04	6.00		
No	542 (94.9%)	8.90	8.00			7.58	6.00		
**Other**				2,841.50	0.94			2,610.50	0.71
Yes	10 (1.8%)	9.20	5.00			6.90	4.00		
No	561 (98.3%)	8.89	8.00			7.56	6.00		

*Statistically significant at p < 0.05.

Depression severity seemed to be most influenced by physical illnesses that were unrelated to COVID-19 ( $\bar{\text{x}}$ = 12.3), followed by inability to access support services ( $\bar{\text{x}}$ = 12.2), and being too emotionally disturbed to focus on academics ( $\bar{\text{x}}$ = 11.6). Anxiety severity was also highest among those with physical illnesses unrelated to COVID-19 ( $\bar{\text{x}}$ = 10.3) and those taking care of a family member who had COVID-19 ( $\bar{\text{x}}$ = 10.3), followed by those who were unable to access support services ( $\bar{\text{x}}$ = 9.9).

#### 3.2.1 Learning difficulty composite

Additionally, since most students experienced more than one challenge to their learning, a learning difficulty composite variable (LD Composite) was created to characterize the cumulative effect of multiple adversities on students’ well-being. For example, an LD Composite score of 3 would indicate a given student experienced three learning difficulties, such as those listed in **Table 3**. Higher LD Composite scores indicate a greater academic burden, potentially posing a more significant barrier to a student’s success.

Notably, most (85.8%) of our participants experienced at least one learning difficulty during the study period. Both depression and anxiety composite scores were much higher in those who had these conditions (depression $\bar{\text{x}}$ = 9.7; anxiety $\bar{\text{x}}$ = 8.1) compared to those who did not (depression $\bar{\text{x}}$ = 4.4; anxiety $\bar{\text{x}}$ = 4.1). In fact, of the factors examined (excluding COVID-19 diagnosis, being required to babysit siblings, and those that selected “other”), all were associated with greater depression or anxiety severity.

Among comorbid cases, the demographic effects on LD Composite were demonstrably more powerful (see **Supplementary Table S3**). Both females and those who selected “other” for their gender had elevated scores ( $\bar{\text{x}}$ = 5.25 and 6.43, respectively, p < 0.05); Asian, Hispanic, and White students ( $\bar{\text{x}}$ = 8.3, 6, and 5), students who lived off-campus ( $\bar{\text{x}}$ =5.4); and students without health insurance ( $\bar{\text{x}}$ = 5.7) (data not shown).

The relationship between LD Composite and total PHQ-9/GAD-7 scores was moderate (ρ = 0.36/0.32, respectively; p < 0.05). The number of learning difficulties diverged with case status. Those with depression and anxiety had the highest amount of difficulties ( $\bar{\text{x}}$ = 5.0), followed closely by those with depression cases ( $\bar{\text{x}}$ = 4.9), anxiety cases ( $\bar{\text{x}}$ = 4.9), and non-cases ( $\bar{\text{x}}$ = 3.2, data not shown).

#### 3.2.2 Learning modalities

Also of interest to the investigators were participants’ experiences with online, Hyflex, and face-to-face learning modalities. Students were able to select one modality, if any, they encountered difficulty with, and of those that responded, 269 (47.1%) selected online, 93 (16.3%) selected Hyflex, 37 (6.5%) selected face-to-face, 75 (13.1%) were unsure, and 24 (4.2%) chose “other.” When depression and anxiety were assessed as a binary variable (yes/no), having trouble with any learning modality was associated with these outcomes independently and jointly (p < 0.05). This trend continued when learning modality’s effect was assessed on depression and anxiety severity (p < 0.05) (**Supplementary Table S4**).

**Figure 2:**
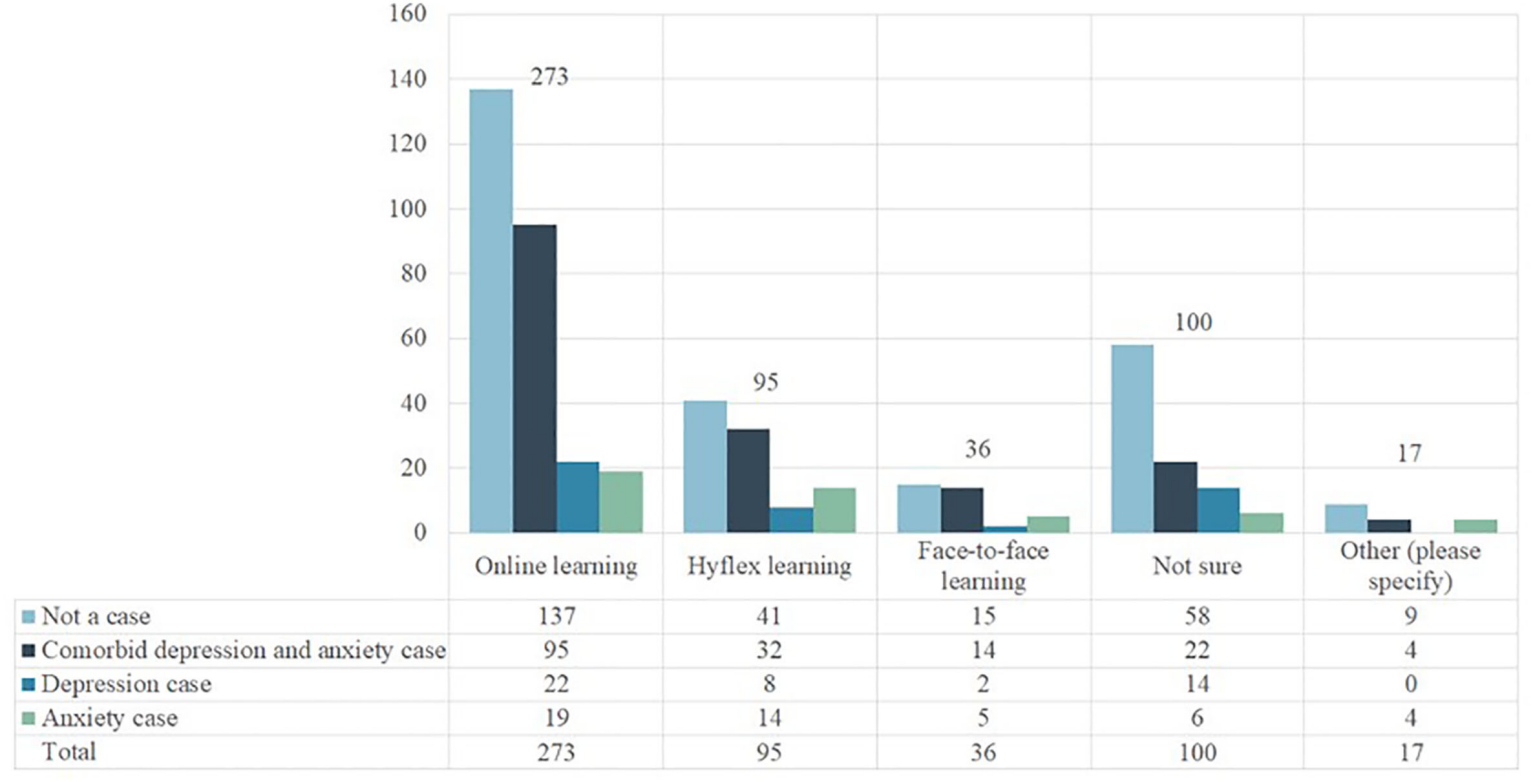
Distribution of depression and anxiety across different learning modalities (n=571).

When depression and anxiety were assessed separately in relation to the learning modalities, only those who could not figure out exactly which modality affected them (the “unsure” group) were associated with anxiety (p < 0.05). Only those who had issues with online issues were associated with anxiety and depression as a comorbid status (p < 0.05). The details are displayed in **Figure 2** and **Supplementary Table S4**.

### 3.3 Factors related to COVID-19

The influence of COVID-19-related factors on depression and anxiety severity is displayed in **Supplementary Table S5**. Vaccinated participants (51%) had higher depression ( $\bar{\text{x}}$ = 9.5) and anxiety ( $\bar{\text{x}}$ = 7.9) scores compared to those who did not. Additionally, those who experienced social stigma associated with COVID-19 infection (29.7%) had higher depression ( $\bar{\text{x}}$ = 12.0) and anxiety ( $\bar{\text{x}}$ = 7.8; p < 0.05) scores, and those with a family history of COVID-19 infection (49%) had an elevated anxiety score ( $\bar{\text{x}}$ = 8.1) as well. We also observed that a family history of COVID-19 infection influenced the LD composite score ( $\bar{\text{x}}$ = 4.4).

As for vaccine uptake intentionality, among the 277 unvaccinated individuals, only 11.6% (32 out of 277) reported intentions to receive the vaccine in the future, while 54% indicated that they would not. The remaining 94 (34%) were undecided or did not know. When intentions were explored with respect to LD composite score, higher scores were observed for those who planned to get the vaccine ( $\bar{\text{x}}$ = 5.2) or were unsure ( $\bar{\text{x}}$ = 4.5) compared to other groups (See **Supplementary Table S6**).

### 3.4 Economic shocks

**Supplementary Table S7** provides information on the consequences of economic obstacles, such as loss of access to employment, food, and housing, on depression and anxiety severity. Among the participants, 179 (31.3%) lost their jobs, 59 (10.3%) lost dependable access to food, and 29 (5.1%) faced housing insecurity. Food and job loss were linked to significantly higher PHQ-9 and GAD-7 scores (p < 0.05), though housing loss was only linked to higher GAD-7 scores (p < 0.05).

The influence of this economic instability on the burden of learning difficulties is also shown in **Supplementary Table S6**. Unemployment, food insecurity, and housing instability were all linked to higher LD Composite scores ( $\bar{\text{x}}$ = 4.7, 6.3, and 5.4, respectively; p < 0.05).

### 3.5 Abuse

Students’ experiences with abuse and their correlating effects on depression and anxiety case status (assessed as binary variables) are presented in **Supplementary Table S8**. Only 304 (53.3%) participants responded to this part of the survey, and of those, only 38 (6.7%) reported some kind of abuse (verbal, emotional, or physical). Among individuals reporting abuse, females were the most affected group (25, 65.8%), Caucasians (23, 58.5%), followed by 20-21-year-olds (20, 52.6%), and Hispanics (9, 23.7%) (data not shown). Furthermore, of those reporting abuse, their parents’ educational level was most often some college (10, 26.3%), a bachelor’s degree (7, 18.4%), or a high school diploma (7, 18.4%), though our study was too underpowered to assess the true relationship between demographic factors and abuse experiences.

While having experienced abuse was associated with case status (p < 0.05), we were unable to assess the relationship between specific forms of abuse (verbal, emotional, or physical) and symptom severity due to the sparsity of data.

### 3.6 Comorbidities

We also investigated the frequency of non-COVID-19 physical illnesses. Among the 135 individuals who reported ever testing positive for COVID-19, 48 (35.6%) had a pre-existing medical condition (e.g., asthma, autoimmune diseases, kidney disease). Of those with a pre-existing condition, the most common were asthma (72.9%), autoimmune disease (12.5%), and arthritis (8.3%) (data not shown).

As seen in **Supplementary Table S9**, while only 9.7% of study participants indicated that they had one of the above physical comorbidities, there seemed to be an association between their illness and case status, as well as their illness and symptom severity (p < 0.05). Additionally, having comorbidity was associated with greater depression and anxiety severity ( $\bar{\text{x}}$ = 11.2 and 10.1, respectively) but not with the burden of learning difficulties, assessed by LD Composite score (**Supplementary Table S6**; p > 0.05).

### 3.7 Information seeking behavior

We also assessed the methods students used to obtain information about the pandemic (**Supplementary Table S10**). Since data was sparse, and the survey only allowed students to choose one category (e.g. TV, newspaper, university emails, social media), we collapsed the responses into four major categories: used traditional media (TV, newspaper, university emails, friends and family, public health authorities), used social media (e.g. Twitter, Facebook, and Snapchat), used neither, and used both.

Among the various sources of information outlets/channels accessed by the participants, a large majority demonstrated a preference for traditional media. However, explaining social media use would have required selecting “other” and inputting a text response. That being said, 455 (79.7%) used traditional media, 47 (8.2%) used social media, 32 (5.6%) used both, and 26 (4.6%) used neither or declined to provide a response.

Using any of the indicated methods of obtaining information about the pandemic at all was associated with both case status and elevated depression ( $\bar{\text{x}}$ = 9) and anxiety ( $\bar{\text{x}}$ = 7.6) severity. In fact, the largest group differences in composite depression and anxiety were between those who did not use any method and those who used both, as well as between those who exclusively employed one method and those who used both (p < 0.05).

A granular analysis of the effect of individual information streams (e.g., TV, friends and family, Facebook, etc.) on composite depression and anxiety scores was also attempted. Use of social media was associated with elevated depression ( $\bar{\text{x}}$ = 10.3) and anxiety ( $\bar{\text{x}}$ = 8.9; data not shown) severity, as well as a higher burden of learning difficulties ( $\bar{\text{x}}$ = 4.9; see **Supplementary Table S6**.)

### 3.8 Coping strategies

**Figure 3:**
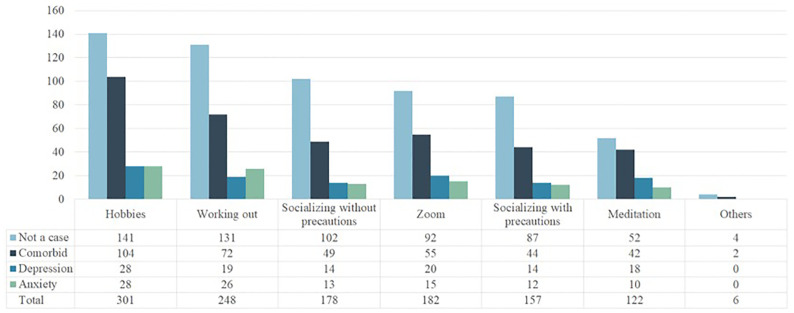
Distribution of depression and anxiety over coping strategies (n = 571).

**Figure 3** visualizes students’ coping methods throughout the pandemic. The most common coping strategies were hobbies (36.4%), exercise (30%), socializing on Zoom or other video conferencing platforms (22%), socializing in person without social distancing and masking (21.5%), and socializing in person with social distancing and masking (19%).

**Supplementary Table S11** displays an analysis of depression and anxiety severity, as well as LD Composite scores, by coping strategy. Elevated depression severity was observed among those who meditated ( $\bar{\text{x}}$ = 10.2) or used hobbies as a coping mechanism ( $\bar{\text{x}}$ = 9.7) compared to their counterparts (p < 0.05). Anxiety severity was higher among those who pursued hobbies as a coping mechanism ( $\bar{\text{x}}$ = 8.1) but lower in those who socialized without pandemic precautions ( $\bar{\text{x}}$ = 6.8). LD Composite scores were also higher in participants who used any of the available coping mechanisms (aside from “other”) (p < 0.05).

## 4 Discussion

The global community observed a 25% increase in the prevalence of depression and anxiety as a result of the pandemic (141), and this effect has also impacted college students (46, 142). Efforts to promote the well-being of students are currently more widespread than ever (23, 58). Still, solidifying and standardizing institutional support has been hindered by ineffective policymaking and a lack of systematic research (143, 144). As communities endure to recover from the impacts of the pandemic, the persistent psychological ramifications of “COVID-era” struggles will persist as students graduate and move into their adult lives. It is, therefore, imperative to actively seek out the ways these experiences have impacted the mental health of students in order to develop well-informed, precise interventions and prevention strategies before another disaster hits the campus communities. If these elements are recognized, and additional research validates our results, academic and community policies could be adjusted to mitigate these situations in the future.

### 4.1 Demographics

Several trends emerged while analyzing demographic data. First, consistent with the existing literature (24, 27, 61, 145, 146), anxiety and depression severity and prevalence demonstrated a gender-based disparity, with a disproportionate burden of these mental health conditions carried by females. For race/ethnicity, the prevalence of depression and anxiety, as well as the comorbid condition, was the highest among Caucasians; Blacks and Hispanics shared a similar burden in these categories. However, when severity was explored as a composite variable, Asians demonstrated the highest severity of these conditions, but due to the small sample size for this category, we have limited inferential ability. Nationally, Asians have the lowest prevalence of mental disorders, followed by Blacks, Latinos, and Whites (147). As for age groups, those that were between the ages of 24-31 had the highest prevalence of depression, but those that were aged 21 had the highest level of anxiety and comorbid condition. Among adults, the highest prevalence of any mental illness is observed among young adults aged 18-25 at 37% of cases. This is followed by individuals aged 26-49, who account for 28.1% of cases, and those aged 50 years and older, who account for 15% of cases (148).

Educational attainment is often used as an indicator of socioeconomic status (149, 150), though it has differing impacts among races. It has been observed that, though higher parental education may be linked to better mental health outcomes in Caucasian students, the same cannot be said for Black students (151). That being said, even among those whose parents have higher levels of educational attainment, this relationship can be mediated by both the interpersonal environment in the family and the circumstances students face on campus (e.g., conflict with friends, heavy course load). We observed that advanced parental education, such as a master’s degree or above, positively impacted our participants’ mental health. This was also seen in students whose parents held an associate degree as well, which suggests a non-linear relationship. Notably, students whose parents had not attained a college degree comprised more than half of the participants who met the diagnostic criteria for anxiety, depression, or both, and these students typically reported more severe symptoms.

The pandemic disproportionately affected low-wage, low-hour workers, who were by nature particularly vulnerable to the economic impacts of shutdowns and stay-at-home orders (8, 152–155). Given that our study represents an institution with a student population of over 50% first-generation students (156), it is likely that our proportion affected by these issues was higher, and the burden of income-related issues was greater (153, 157). Notably, African Americans and American Indians also had considerably higher depression and anxiety scores, which could be due to the effects of lasting systemic racism (154, 158–160).

Traditional students also seemed to have greater depression and anxiety severity in our study. Since they typically begin their college education immediately after graduating from high school, they are usually younger and rely on their parents for financial support. Though non-traditional students are usually older and juggle multiple roles, responsibilities, and financial commitments, traditional students most often do not have the life experience or financial stability to absorb existential stressors such as the pandemic (15).

### 4.2 Academics

#### 4.2.1 Learning difficulties

Research has consistently linked intrinsic motivation with academic success (161–164). Among the factors explored, motivation was identified as one of the primary obstacles to academic success among participants with depression and anxiety. However, it is important to recognize that there could be a bidirectional relationship between the mental health conditions explored and motivation, which can impact learning. For instance, anxiety and depression might have led to a decline in motivation, and in turn, lockdowns and limited academic experiences might have caused a fear that the academic year has been lost (57). In the same vein, students who were more exposed to information about COVID-19 may have been distressed by the constant stream of negative news (165–168), which in turn could have resulted in a lack of motivation.

Another noted barrier to learning is the high cost of acquiring and becoming proficient in using modern technology, especially e-learning platforms (65, 169, 170). It is evident that the transition to online education brought about fresh hurdles for achieving academic success amidst the pandemic (65, 170), as just under 20% of our participants did not have a good quality microphone or camera on their computer. Moreover, study participants returning to rural regions after college closures with reduced access to the internet (35.2%) seemed to have experienced exacerbated depression and anxiety symptoms, falling in line with existing literature during this time (153, 171).

There were also variations by race. On the lower extreme, American Indians and Caucasians enjoyed fewer learning difficulties, with correlating depression and anxiety scores in a sub-clinical range. In contrast, Asian and Hispanic students encountered more learning difficulties, which were associated with clinical levels of depression and anxiety. Our findings in this regard were comparable with previous literature observing racial disparities in academic performance (59, 61, 154, 172, 173).

#### 4.2.2 Learning modalities

Previously, the transition to online learning has been linked to an increase in the symptoms of depression and anxiety (61, 64, 174–177). Students, now required to keep track of lectures, assignments, and exams in a remote environment, suffered as external support fell away. Essential resources, such as a stable internet connection and privacy (178), were suddenly barred from access, and students and professors alike struggled to keep afloat.

Initially, when exploring learning difficulties as a binary variable, difficulty with learning in any modality seemed to be linked with anxiety cases, depression cases, and comorbid depression and anxiety cases. Additional tests exploring composite depression and anxiety as continuous variables corroborated these results and further highlighted that those with a higher level of depression and anxiety severity experienced learning difficulties, most notably with online learning. However, upon examining the composite depression and anxiety scores of those who had trouble with any learning modality, rather than just the sample at large, it seems that there is no significant difference among any of the available options (online, Hyflex, face-to-face, not sure, or other). In fact, among those who were “unsure,” the prevalence of anxiety cases was lower, and among those who struggled with online learning, the prevalence of comorbid depression and anxiety was also lower. It seems that the association only exists among the sample at large, and the true cause of the increased severity was having trouble with any learning modality at all. This may suggest that either the shift to online learning was responsible for the distress experienced by college students impacting their mental health or that students who were already struggling academically may have had a more difficult time transitioning to online learning platforms during the pandemic.

### 4.3 COVID-19

When universities closed and students returned home in the early spring of 2020, the prevalence of COVID-19 was not fully known. It was, however, becoming apparent that younger individuals usually experienced less severe symptoms, and a significant portion were asymptomatic but still infectious (179). In our study, roughly 11% of the participants were identified as having ever contracted COVID-19, but no significant correlation was established between those diagnosed and undiagnosed individuals in relation to mental health conditions. Nonetheless, other experiences related to the pandemic appeared to have an impact on their well-being.

Though vaccines were made available just before the end of 2020, it was not until April of 2021 that individuals aged 16 and older were eligible to receive them (180, 181). Our data was collected in the summer of 2021, by which time many students had obtained the vaccine. We found that those who had received the vaccine (51%) exhibited higher rates of depression and anxiety compared to those who had not been vaccinated. Although the literature suggests that anxious and depressed adults tend to be more susceptible to vaccine misinformation and less likely to undergo vaccination, our study did not investigate this relationship (182–184).

While we cannot ascertain causality, other influential factors might have been uncertainty surrounding the vaccination (particularly with respect to its side effects and efficacy), as well as a higher risk perception of the pandemic in general (83, 185–187). It has also been found that those who researched the vaccines using credible scientific sources on social media were more likely to vaccinate than those who did not (188). Unfortunately, rife misinformation led to vaccine hesitancy (30, 189, 190), and college students were no exception (191–193), including our study population.

We also assess the impact of stigma with COVID-19 infection on mental health outcomes. In general, individuals who contracted COVID-19 and exhibited symptoms, as well as their close contacts were often subjected to suspicion and intimidation (194), with Asian Americans also being the target of disproportionate scrutiny and violence (195, 196). Previous outbreaks have regrettably established the foundation for shame, othering, and distance from individuals who contracted illnesses (197–200), and in skewing the way the public views risks, stigma has led to widespread panic and unequal distribution of healthcare resources (199). In our study, students who encountered stigma due to COVID-19 infection exhibited greater clinical scores for anxiety and depression as compared to those who did not experience stigma during the study period.

### 4.4 Economic shocks

The effects of economic difficulties on mental health and the ability to learn are well documented (158, 201), and this trend continued into the pandemic (202–204). The economic impact of the COVID-19 pandemic in early 2020 was severe, as when the economy was shut down to prevent its spread, the United States’ uniquely frayed social safety net did not protect its citizens (205–207), nor did institutions have the capability to protect their students.

To students, university closures meant that protective factors—such as dependable access to food, housing, and employment—were also shuttered away (55,134). Students were significantly impacted due to the confluence of their and their families’ economic precarity (86), as they had to contend not only with the absurdly high cost of tuition, room, and board (135) but also the disparate effects of the job market (63, 101, 152, 153).

Our participants themselves were most commonly struck by job loss, though food and housing insecurity were reported by substantially fewer. Job loss seemed to have the greatest influence on mental illness severity, but the smallness of the sample might have obscured the true impact of greater financial instability. It stands to reason that those experiencing more significant instabilities might lack the support to continue their schooling (208, 209). The limited data from students who lost reliable access to either food or housing may indicate that greater economic hardship leads to withdrawal and that our participants were best able to absorb the loss of a job rather than a loss of food or housing.

### 4.5 Abuse

The presence of abuse in students’ lives creates an environment that easily gives rise to mental illness (9, 210, 211). Though students are less likely to be forthcoming with their experiences regarding abuse (212–215), 42 (5.1%) of our participants indicated that they experienced abuse during the study period. Overwhelmingly, they were reported by either undergraduates, females, or Caucasian students, which is fairly in line with existing research indicating that Caucasian women are more likely to report abuse (216–218).

Given their age and economic status, pandemic-era campus closures left many students vulnerable in their homes (101, 204, 219, 220). Of our participants who were abused when they returned home, almost all experienced verbal abuse, several experienced emotional abuse, and two experienced physical abuse. Although the smallness of the sample makes it difficult to interpret the associations with high confidence, the most robust results are obtained by the binary “experienced abuse”/”did not.” The majority of abuse survivors met the clinical threshold for anxiety or depression, and the plurality met the threshold for both.

Few articles examine the immediate impacts of abuse on college students, but in general, abuse seems to be most profoundly correlated with depression and posttraumatic stress (10, 221–224). Further research should examine the short- and long-term effects of exposure to abuse during this period.

### 4.6 Comorbidities

A bidirectional relationship exists between chronic illness and mental health (225–228). While sparseness of our data restricts the inferences that can be confidently drawn regarding the impact of comorbidities on mental health during the pandemic itself, we are able to corroborate other research that suggests an exacerbation of this relationship during this time period (229).

Of our participants that reported comorbidities, the overwhelming majority (69.6%) had asthma. Although asthma has been associated with anxiety and depression (230), recent findings indicate an increased risk of anxiety, stress, and depression among individuals with asthma during the pandemic (231, 232). It’s likely that, understanding their increased vulnerability to severe COVID-related illness (and having faced that vulnerability daily for almost a year and a half), those with respiratory comorbidities experienced anxiety and depression much more easily.

The presence of underlying comorbidities such as cardiovascular and autoimmune disease can potentially increase the risk of contracting and developing more lethal forms of COVID-19 (233, 234). Among the fairly young adult participants in our study, around 10% were found to have a high-risk comorbidity. These conditions could have exacerbated or been exacerbated by COVID-19 infection (233, 234).

### 4.7 Information channels

Social media usage became an essential source of pandemic-related information for the majority of the US population during the pandemic. Many Americans relied on social media for pandemic-related information to some degree, but due to prevalent misinformation and skepticism, very few verified this information with a healthcare provider (188). Social media use has, therefore, been a double-edged sword (235), as while it has allowed its users to connect, express themselves, and seek information (236), it has also fostered feelings of anger, helplessness, and distrust (166, 167, 235–238). These findings, though, require more scrutiny, as social media use is deeply personal, and it is difficult to make broad generalizations (239, 240).

Our study participants employed various strategies to access information about the pandemic. Most commonly, they used university emails (44.1%), TV news (19.4%), and social media (10%). Among those who used social media, Facebook (31.7%) was the most commonly used platform, followed by Twitter (29.1%) and TikTok (11.4%). With the exception of television news, it does seem that the frequency of response is somewhat correlated with a higher average composite score, which does underline the flaw in the way the survey was formatted. Since the survey did not allow students to select more than one way of obtaining pandemic-related information, more robust results could have been obtained if students could select all the ways they sought information.

Some results must be cautiously interpreted due to the small sample sizes in the different categories of information-seeking behaviors. However, after collapsing the categories, using any method at all (traditional or social) to obtain information about the pandemic was associated with elevated anxiety and a higher burden of learning difficulties. This could imply that soliciting any information about the pandemic caused distress or that those already distressed by the pandemic actively sought out this information.

However, the degree to which the participants interact with the information seems to better predict distress. There is a small jump in anxiety and depression severity between those who did not solicit any information and those who only used one type of media, but a sharp increase in both is observed in those who used both types of media. It seems that the variety and duration of time spent receiving pandemic-related information is correlated with increased symptoms of depression and anxiety (168, 241). Though there is little research examining the exposure to information by platform, there is a broad consensus that excessive use of social media leads to negative mental health outcomes (166, 167, 237).

### 4.8 Coping strategies

In light of the COVID-19 pandemic, research indicates that coping mechanisms such as positive reframing, humor, and acceptance can improve mental health outcomes (242, 243). In our study population, students used a variety of methods to cope with stress throughout the pandemic. Across all participants, the most popular coping strategies included hobbies, working out, and socializing. Among those who reported having mental health conditions, there was a slight preference for socializing with precautions, such as social distancing and masking, over socializing without. Generalized findings, though, are in line with previous research regarding college students’ coping strategies during this time (244–248).

Notably, there was a preference for using hobbies and meditation in those with depression. This coincides with the rapid growth of wellness and fitness apps, such as Calm, Headspace, and Fitbit in 2020 (249, 250). While we are unable to make inferences about the direction of causality [even if recent literature indicates mindfulness may protect against more severe expressions of mental illness (251, 252)], it does seem that either those who have more severe symptoms are more likely to use coping strategies like meditation or hobbies, or these methods are less efficient at reducing symptoms.

### 4.9 Strengths and limitations

#### 4.9.1 Limitations

Our investigation was primarily focused on students attending a public university located in central Texas with satellite campuses in north Texas. While the university has representation from almost every county in the state (253), it is important to note that the conclusions drawn from our research may not be applicable to public universities in other states, private institutions, Historically Black Colleges and Universities (HBCUs), or Hispanic-Serving Institutions (HSIs) (65). The university has, however, recently launched an initiative to become an HSI (254).

Some methodological and sampling limitations could influence the generalizability of our findings because of their cross-sectional design. For this reason, we were not able to evaluate critical temporal relationships but were only able to explore associations. Furthermore, study data were obtained through a survey. The anonymity of the survey may have helped to somewhat alleviate the effects of social desirability bias (255).

On a similar note, data missingness was a limitation of our study. Due to the use of an online survey, participants could answer or ignore questions at their own discretion; these ignored items resulted in missing data within our study. We initially had 828 survey respondents, but 377 responses were removed due to missing data, resulting in a final sample of 571 participants. Furthermore, any remaining missing data within the study sample were handled using pairwise deletion. Although deletion strategies when handling missing data can result in a loss of statistical power (Osborne, 2013), our final sample size of 571 participants potentially mitigated this issue. However, data missingness can still cause results to potentially become biased (Peugh and Enders, 2004), and so this limitation should be noted.

It was also not possible to examine some of the variables with high confidence, as participants not only needed to have completed both the PHQ-9 and GAD-7 in full but also to have answered survey questions appropriately. Furthermore, some variables were created after the study period had concluded (e.g., combining different types of difficulty with learning modalities into yes/no categories). While these variables allowed us to do more granular analysis, our confidence in such inferences is limited. This primarily pertains to our analyses of abuse, physical comorbidities, and information channels, but it also extends to more in-depth looks at economic shocks.

Another factor that is important to consider is that our research might have captured prevalent cases of depression and anxiety that existed prior to the pandemic. Existing research implies that up to 60% of those with mental health disorders diagnosed during the pandemic had persistent symptoms up to two years after the initial assessment (256). Therefore, some of the cases may not be directly attributable to the pandemic stressors, even though their severity may have increased.

As mentioned, job losses were reported by more study participants in our study than loss of food and housing, possibly due to its limited impact on a student’s ability to continue schooling. Furthermore, employment inquiries that are particular to each job, including working on-campus and off-campus, were not part of the survey, which restricts our ability to draw conclusions about the influence of job loss per location and its independent impact on mental health. However, it is probable that first-generation students and transfer students who relied on income from wage and salary jobs were more impacted by the pandemic than Pell Grant recipients (153). Unfortunately, we could not analyze these factors due to the lack of data.

Many of our college students come from rural communities (257). When colleges and universities transitioned to remote operations during the pandemic, rural poor and working-class students encountered geographic and social class obstacles related to the accessibility of high-speed internet and dependable technology (258). This could have impacted learning for some of the students who resided in rural areas. Unfortunately, we could not examine the association between rurality and learning difficulties, which could have affected the mental health of these students. Furthermore, each learning difficulty impacted mental health in a different way. While the cumulative effect of each one potentially influenced both depression and anxiety, the LD Composite remains a more generalized summary of a student’s experience.

There is conflicting evidence that the variety of social media and duration of time spent receiving pandemic-related information has increased symptoms of depression and anxiety (168, 240, 241, 259–261). However, several studies conducted in the pre-pandemic era have identified time spent, activity, and social media addiction as potential factors affecting anxiety and depression (240). Due to the absence of data on the frequency and duration of social media use in the survey, we could not examine the relationships of such nuances on the outcomes of interest. We were also unable to see the impact of social media type (e.g., Facebook, Instagram) on the severity of outcomes due to sparse data.

#### 4.9.2 Strengths

Failure to recognize or accept mental health symptoms, coupled with insufficient treatment, are prevalent issues among college students (15). Our primary objective was to assess depression and anxiety, which are frequently reported conditions among college students seeking mental health assistance on university grounds (23). These two disorders have far-reaching consequences that can potentially impact their lives well into adulthood and beyond. We aimed to offer insight into the elements that could exacerbate these conditions. To adequately address these factors, it is crucial to consider both individual and environmental influences on mental health within educational settings. Our comprehensive research provides an in-depth analysis of a broad range of factors that likely influenced college students’ mental health outcomes. In addition, it offers valuable insights and suggestions for generating hypotheses to explore these associations further. This approach deepens our understanding of the various mediators and moderators involved many of which are frequently overlooked in similar research, thus offering a more refined perspective on these complex relationships and paving the way for future studies.

The significant rise in the incidence of depression and anxiety among young people in the United States and other nations since the beginning of the pandemic is a matter that merits attention and examination, as these conditions could potentially lead to suicidal and self-harming behaviors (141, 262, 263). This study aimed to address this critical issue by gathering vast data from students during the pandemic. This enabled us to capture timely information and identify factors that exacerbated various aspects of students’ college life. This was enabled by our use of a cross-sectional design, which allowed us to generate a great number of hypotheses and observations. Although the sample sizes of some of our analyses were relatively small to tease out granular details, we have identified several noteworthy findings that warrant further exploration with larger samples.

By utilizing convenience sampling, we could provide a snapshot of students’ mental health during the summer of 2021. Most critically, it may provide an idea of what the confluence of the many effects of the pandemic (lockdowns, interaction with information, socioeconomic status, etc.) may produce in students. In doing so, the university and higher educational institutions at large will be able to anticipate the needs of their students and understand the burden of the trauma they experience. Furthermore, we replicated some of the important associations using validated questionnaires to assess anxiety and depression in our study, illustrating the pervasive nature of mental illness and its associated covariates among college students (8, 47, 171, 233, 264, 265). This phenomenon could be further intensified during periods of large-scale disasters such as COVID-19. To identify the precise factors that exacerbate existing mental health conditions and contribute to the emergence of new ones among college students during disasters, longitudinal studies are essential.

## 5 Conclusion

This extensive study sheds light on the various factors connected to the pandemic and their effects on the mental well-being of university students. By identifying potential influencers as well as mediators and moderators, this study paves the way for more targeted and effective mental health support in academic settings during large-scale health crises. While it is widely acknowledged that disasters can profoundly impact mental health and academic performance, the study recognizes that these effects may not be uniform across the student population. Instead, the research suggests that the extent and nature of these impacts may vary considerably based on a complex interplay of individual characteristics and contextual factors. These factors may include, but are not limited to, personal resilience, comorbidities, stressors associated with the learning environment, social connectivity access, basic needs, coping strategies, access to mental health services, and additional relevant variables. Since our study was a cross-sectional study, future studies can examine the long-term impact of the implications of stressors encountered by students, such as the abrupt shift to online learning, social disconnection, and abuse experienced during the pandemic, to investigate the long-term effects. This will emphasize the need for continued efforts to address mental health in student populations in the post-COVID-19 era and better prepare us for the future pandemics.

Due to the diverse and multidimensional aspects of pandemic-induced stressors for students, this research also provides insights to educational institutions to enhance their preparedness for future emergencies and provide mental health support to their students. By examining the key factors influencing mental health, colleges, and universities can enhance their ability to address students’ psychological well-being during and after crisis situations. This approach may potentially mitigate long-term mental health consequences among students during and after their collegiate experience. Although accurately predicting the next pandemic is challenging, analyzing and deriving insights from historical efforts for preparedness and response may be an effective strategy to address students’ psychological well-being challenges during future health emergencies.

## Data Availability

The datasets analyzed for this study can be found in Mendeley. Gandhi, Subi (2024), “COVID-19 and Mental Health”, Mendeley Data, V1, doi: 10.17632/htb8s666tm.1.
