# Supplementary material for: Unmasking the impact of COVID-19 on the mental health of college students: a cross-sectional study
Source: Front Psychiatry. 2024 Nov 18;15:1453323. doi: 10.3389/fpsyt.2024.1453323 (PMC11608972; doi:10.3389/fpsyt.2024.1453323)
Supplement: Supplementary file 1 [file Table1.docx]

| **Supplementary Table 1. Demographic and Other Characteristics Breakdown of Participants by Anxiety and Depression Severity Categories (N = 571)** | | | | | | | | | | | | | | |
| --- | --- | --- | --- | --- | --- | --- | --- | --- | --- | --- | --- | --- | --- | --- |
|  | |  |  | **Depression Severity (PHQ-9)** |  |  |  |  |  |  | **Anxiety Severity**  **(GAD-7)** |  |  |  |
| **Category** | | **No Depression**  **(0-4)** | **Mild Depression**  **(5-9)** | **Moderate Depression**  **(10-14)** | **Moderately Severe Depression**  **(15-19)** | **Severe Depression**  **(20-27)** |  |  | **No - Minimal Anxiety**  **(0-4)** | **Mild Anxiety**  **(5-9)** | **Moderate Anxiety**  **(10-14)** | **Severe Anxiety**  **(15-21)** |  |  |
| **N** | | **194** | **152** | **87** | **84** | **54** |  |  | **211** | **162** | **115** | **83** |  |  |
|  | |  |  |  |  |  | **V** | **p** |  |  |  |  | **V** | **p** |
| **Gender** |  |  |  |  |  |  | 0.17 | 0.05* |  |  |  |  | 0.18 | < 0.01 |
| Male | 145 (25.4%) | 53 | 41 | 18 | 20 | 13 |  |  | 68 | 32 | 31 | 14 |  |  |
| Female | 411 (72.0%) | 139 | 107 | 62 | 64 | 39 |  |  | 143 | 123 | 79 | 66 |  |  |
| Other | 15 (2.6%) | 2 | 4 | 7 | 0 | 2 |  |  | 0 | 7 | 5 | 3 |  |  |
| Missing | 0 (0.0%) | 0 | 0 | 0 | 0 | 0 |  |  | 0 | 0 | 0 | 0 |  |  |
| **Age** |  |  |  |  |  |  | 0.14 | 0.03* |  |  |  |  | 0.12 | 0.17 |
| 18 | 42 (7.4%) | 16 | 10 | 8 | 5 | 3 |  |  | 20 | 12 | 7 | 3 |  |  |
| 19 | 77 (13.5%) | 23 | 28 | 7 | 13 | 6 |  |  | 26 | 23 | 19 | 9 |  |  |
| 20 | 81 (14.2%) | 30 | 19 | 9 | 15 | 8 |  |  | 27 | 22 | 22 | 10 |  |  |
| 21 | 74 (13.0%) | 19 | 21 | 8 | 14 | 12 |  |  | 20 | 21 | 21 | 12 |  |  |
| 22-23 | 81 (14.2%) | 25 | 19 | 12 | 15 | 10 |  |  | 29 | 20 | 16 | 16 |  |  |
| 24-31 | 84 (14.7%) | 23 | 20 | 20 | 13 | 8 |  |  | 25 | 27 | 15 | 17 |  |  |
| 32+ | 94 (16.5%) | 41 | 26 | 20 | 4 | 3 |  |  | 46 | 25 | 13 | 10 |  |  |
| Missing | 38 (6.7%) | 17 | 9 | 3 | 5 | 4 |  |  | 18 | 12 | 2 | 6 |  |  |
| **Race** |  |  |  |  |  |  | 0.14 | < 0.01* |  |  |  |  | 0.12 | 0.13 |
| Caucasian | 355 (62.2%) | 135 | 97 | 52 | 44 | 27 |  |  | 134 | 105 | 69 | 47 |  |  |
| African American | 74 (13.0%) | 17 | 15 | 14 | 21 | 8 |  |  | 22 | 17 | 25 | 11 |  |  |
| Hispanic | 103 (18.0%) | 26 | 32 | 15 | 16 | 14 |  |  | 37 | 29 | 18 | 19 |  |  |
| Asian | 7 (1.2%) | 2 | 2 | 0 | 0 | 3 |  |  | 3 | 1 | 0 | 3 |  |  |
| American Indian | 5 (0.9%) | 1 | 1 | 1 | 2 | 0 |  |  | 1 | 2 | 1 | 1 |  |  |
| East Indian | 2 (0.4%) | 2 | 0 | 0 | 0 | 0 |  |  | 1 | 0 | 1 | 0 |  |  |
| Other | 24 (4.2%) | 11 | 5 | 5 | 1 | 2 |  |  | 13 | 8 | 1 | 2 |  |  |
| Missing | 1 (0.2%) | 0 | 0 | 0 | 0 | 0 |  |  | 0 | 0 | 0 | 0 |  |  |
| **Parent's level of education** |  |  |  |  |  |  | 0.13 | 0.02* |  |  |  |  | 0.10 | 0.46 |
| Some high school | 65 (11.4%) | 11 | 14 | 15 | 13 | 12 |  |  | 14 | 17 | 19 | 15 |  |  |
| High school | 96 (16.8%) | 31 | 30 | 12 | 15 | 8 |  |  | 38 | 24 | 20 | 14 |  |  |
| Some college | 119 (20.8%) | 41 | 34 | 18 | 16 | 10 |  |  | 43 | 39 | 19 | 18 |  |  |
| Associate’s degree | 52 (9.1%) | 18 | 11 | 5 | 14 | 4 |  |  | 22 | 13 | 10 | 7 |  |  |
| Bachelor’s degree | 15 (2.6%) | 60 | 36 | 28 | 20 | 10 |  |  | 61 | 44 | 31 | 18 |  |  |
| Master’s degree | 67 (11.7%) | 24 | 23 | 8 | 4 | 8 |  |  | 23 | 22 | 14 | 8 |  |  |
| Doctorate degree | 11 (1.9%) | 7 | 1 | 0 | 2 | 1 |  |  | 6 | 1 | 2 | 2 |  |  |
| Missing | 146 (25.6%) | 2 | 3 | 1 | 0 | 1 |  |  | 4 | 2 | 0 | 1 |  |  |
| **Non-traditional status** |  |  |  |  |  |  | 0.18 | < 0.01* |  |  |  |  | 0.09 | 0.21 |
| Yes | 116 (20.3%) | 57 | 45 | 38 | 12 | 14 |  |  | 68 | 47 | 25 | 26 |  |  |
| No | 396 (69.4%) | 134 | 103 | 49 | 70 | 40 |  |  | 138 | 113 | 89 | 56 |  |  |
| Missing | 59 (10.3%) | 3 | 4 | 0 | 2 | 0 |  |  | 5 | 2 | 1 | 1 |  |  |
| **First-generation students** |  |  |  |  |  |  | 0.08 | 0.48 |  |  |  |  | 0.06 | 0.53 |
| Yes | 254 (44.5%) | 84 | 75 | 39 | 37 | 19 |  |  | 94 | 79 | 48 | 33 |  |  |
| No | 316 (55.3%) | 110 | 77 | 47 | 47 | 35 |  |  | 117 | 83 | 66 | 50 |  |  |
| Missing | 1 (0.2%) | 0 | 0 | 1 | 0 | 0 |  |  | 0 | 0 | 1 | 0 |  |  |
| **Undergraduate classification** |  |  |  |  |  |  | 0.07 | 0.93 |  |  |  |  | 0.09 | 0.31 |
| Freshman | 45 (7.9%) | 15 | 13 | 7 | 6 | 4 |  |  | 21 | 11 | 8 | 5 |  |  |
| Sophomore | 91 (15.9%) | 27 | 30 | 10 | 17 | 7 |  |  | 29 | 27 | 26 | 9 |  |  |
| Junior | 143 (25.0%) | 50 | 33 | 20 | 23 | 17 |  |  | 50 | 40 | 35 | 18 |  |  |
| Senior | 172 (30.1%) | 58 | 41 | 27 | 27 | 19 |  |  | 63 | 44 | 32 | 33 |  |  |
| Missing | 120 (21.0%) | 44 | 35 | 23 | 11 | 7 |  |  | 48 | 40 | 14 | 18 |  |  |
| **Degree level** |  |  |  |  |  |  | 0.08 | 0.61 |  |  |  |  | 0.08 | 0.22 |
| Undergraduate | 409 (71.6%) | 136 | 103 | 60 | 66 | 44 |  |  | 145 | 109 | 95 | 60 |  |  |
| Graduate | 144 (25.2%) | 50 | 45 | 22 | 17 | 10 |  |  | 58 | 47 | 18 | 21 |  |  |
| Postgraduate | 14 (2.5%) | 6 | 3 | 4 | 1 | 0 |  |  | 7 | 3 | 2 | 2 |  |  |
| Other | 3 (0.5%) | 1 | 1 | 1 | 0 | 0 |  |  | 1 | 2 |  |  |  |  |
| Missing | 1 (0.2%) | 1 | 0 | 0 | 0 | 0 |  |  | 0 | 1 | 0 | 0 |  |  |
| **College** |  |  |  |  |  |  | 0.10 | 0.42 |  |  |  |  | 0.12 | 0.06 |
| Agricultural and Environmental Sciences | 93 (16.3%) | 25 | 29 | 17 | 11 | 11 |  |  | 26 | 33 | 16 | 18 |  |  |
| Business | 106 (18.6%) | 40 | 25 | 17 | 13 | 11 |  |  | 47 | 23 | 23 | 13 |  |  |
| Education | 92 (16.1%) | 27 | 21 | 15 | 21 | 8 |  |  | 28 | 26 | 19 | 19 |  |  |
| Health Sciences and Human Services | 123 (21.5%) | 49 | 36 | 14 | 15 | 9 |  |  | 48 | 43 | 21 | 11 |  |  |
| Liberal and Fine Arts | 79 (13.8%) | 29 | 17 | 9 | 16 | 8 |  |  | 34 | 13 | 19 | 13 |  |  |
| Science and Technology | 77 (13.5%) | 23 | 24 | 15 | 8 | 7 |  |  | 27 | 24 | 17 | 9 |  |  |
| Missing | 1 (0.2%) | 1 | 0 | 0 | 0 | 0 |  |  | 1 | 0 | 0 | 0 |  |  |
| **Campus Residence** |  |  |  |  |  |  | 0.12 | 0.10 |  |  |  |  | 0.13 | 0.02* |
| On-campus | 181 (31.7%) | 54 | 47 | 25 | 37 | 18 |  |  | 61 | 47 | 50 | 23 |  |  |
| Off-campus | 389 (68.1%) | 140 | 105 | 62 | 47 | 35 |  |  | 150 | 115 | 64 | 60 |  |  |
| Missing | 1 (0.2%) | 0 | 0 | 0 | 0 | 1 |  |  | 0 | 0 | 1 | 0 |  |  |
| **Health Insurance** |  |  |  |  |  |  | 0.10 | 0.22 |  |  |  |  | 0.04 | 0.81 |
| Yes | 453 (79.3%) | 162 | 120 | 67 | 60 | 44 |  |  | 172 | 126 | 91 | 64 |  |  |
| No | 117 (20.5%) | 32 | 31 | 20 | 24 | 10 |  |  | 39 | 35 | 24 | 19 |  |  |
| Missing | 1 (0.2%) | 0 | 1 | 0 | 0 | 0 |  |  | 0 | 1 | 0 | 0 |  |  |
| *Statistically significant at p < 0.05 | | | |  |  |  |  |  |  |  |  |  |  |  |
