# Supplementary material for: Unmasking the impact of COVID-19 on the mental health of college students: a cross-sectional study
Source: Front Psychiatry. 2024 Nov 18;15:1453323. doi: 10.3389/fpsyt.2024.1453323 (PMC11608972; doi:10.3389/fpsyt.2024.1453323)
Supplement: Supplementary file 2 [file Table2.docx]

| **Supplementary Table 2. Characteristics of Participants with Both Depression and Anxiety (N = 177)** | | | | | | | | | |
| --- | --- | --- | --- | --- | --- | --- | --- | --- | --- |
| **Variable** |  | **Composite PHQ-9 (Depression) Score** | | | | **Composite GAD-7 (Anxiety) Score** | | | |
|  | **N = 177** | **x̄** | **M** | **KW/MW** | **p** | **x̄** | **M** | **KW/MW** | **p** |
| **Gender** |  |  |  | 1.38 | 0.50 |  |  | 1.55 | 0.46 |
| Male | 41 (23.2%) | 17.68 | 18.00 |  |  | 14.44 | 14.00 |  |  |
| Female | 129 (72.9%) | 17.22 | 17.00 |  |  | 15.10 | 14.00 |  |  |
| Other | 7 (4.0%) | 15.71 | 13.00 |  |  | 13.57 | 13.00 |  |  |
| **Age** |  |  |  | 7.38 | 0.29 |  |  | 4.27 | 0.64 |
| 18 | 9 (5.1%) | 14.67 | 12.00 |  |  | 13.11 | 12.00 |  |  |
| 19 | 23 (13.0%) | 17.00 | 17.00 |  |  | 14.22 | 14.00 |  |  |
| 20 | 27 (15.3%) | 17.85 | 18.00 |  |  | 14.48 | 14.00 |  |  |
| 21 | 33 (18.6%) | 18.06 | 18.00 |  |  | 14.79 | 14.00 |  |  |
| 22-23 | 31 (17.5%) | 17.55 | 17.00 |  |  | 15.77 | 15.00 |  |  |
| 24-31 | 29 (16.4%) | 16.69 | 16.00 |  |  | 14.93 | 15.00 |  |  |
| 32+ | 16 (9.0%) | 15.75 | 14.00 |  |  | 15.50 | 15.50 |  |  |
| **Race** |  |  |  | 7.59 | 0.18 |  |  | 6.91 | 0.23 |
| Caucasian | 100 (56.5%) | 16.72 | 16.00 |  |  | 14.69 | 14.00 |  |  |
| African American | 35 (19.8%) | 17.11 | 17.00 |  |  | 14.34 | 14.00 |  |  |
| Hispanic | 35 (19.8%) | 18.54 | 17.00 |  |  | 15.29 | 15.00 |  |  |
| Asian | 3 (1.7%) | 22.67 | 21.00 |  |  | 19.33 | 21.00 |  |  |
| American Indian | 1 (0.6%) | 18.00 | 18.00 |  |  | 18.00 | 18.00 |  |  |
| East Indian | 0 (0.0%) |  |  |  |  |  |  |  |  |
| Other | 3 (1.7%) | 16.67 | 17.00 |  |  | 17.67 | 19.00 |  |  |
| **Parent/Guardian’s Education Level** |  |  |  | 5.75 | 0.45 |  |  | 4.44 | 0.62 |
| Some high school | 33 (18.6%) | 18.21 | 18.00 |  |  | 14.70 | 14.00 |  |  |
| High school | 27 (15.3%) | 17.52 | 18.00 |  |  | 15.41 | 15.00 |  |  |
| Some college | 36 (20.3%) | 16.94 | 16.00 |  |  | 15.42 | 14.50 |  |  |
| Associate’s degree | 16 (16.0%) | 17.63 | 18.00 |  |  | 14.88 | 14.00 |  |  |
| Bachelor’s degree | 45 (25.4%) | 16.11 | 16.00 |  |  | 13.89 | 14.00 |  |  |
| Master’s degree | 16 (9.0%) | 18.00 | 18.50 |  |  | 15.50 | 15.00 |  |  |
| Doctorate degree | 3 (1.7%) | 18.33 | 17.00 |  |  | 16.00 | 14.00 |  |  |
| **Non-Traditional Students** |  |  |  | 2879.00 | 0.76 |  |  | 2277.00 | 0.07 |
| Yes | 42 (24.7%) | 17.29 | 16.50 |  |  | 15.83 | 15.50 |  |  |
| No | 133 (75.1%) | 17.26 | 17.00 |  |  | 14.58 | 14.00 |  |  |
| **First Generation Students** |  |  |  | 3988.50 | 0.43 |  |  | 3890.50 | 0.62 |
| Yes | 71 (40.1%) | 17.01 | 17.00 |  |  | 14.75 | 14.00 |  |  |
| No | 105 (59.3%) | 17.50 | 18.00 |  |  | 15.00 | 14.00 |  |  |
| **Undergraduate Classification** |  |  |  | 1.10 | 0.78 |  |  | 3.55 | 0.32 |
| Freshman | 11 (6.2%) | 15.91 | 16.00 |  |  | 14.55 | 13.00 |  |  |
| Sophomore | 27 (15.3%) | 17.37 | 17.00 |  |  | 13.97 | 14.00 |  |  |
| Junior | 50 (28.2%) | 17.36 | 17.50 |  |  | 14.46 | 14.00 |  |  |
| Senior | 63 (35.6%) | 17.36 | 18.00 |  |  | 15.51 | 15.00 |  |  |
| **Degree Level** |  |  |  | 2.58 | 0.28 |  |  | 1.24 | 0.54 |
| Undergraduate | 142 (80.2%) | 17.37 | 17.00 |  |  | 14.71 | 14.00 |  |  |
| Graduate | 32 (18.1%) | 17.19 | 16.50 |  |  | 15.59 | 15.00 |  |  |
| Postgraduate | 3 (1.7%) | 13.33 | 12.00 |  |  | 15.33 | 15.00 |  |  |
| Other | 0 (0.00%) |  |  |  |  |  |  |  |  |
| **College** |  |  |  | 2.33 | 0.80 |  |  | 2.06 | 0.84 |
| Agricultural and Environmental Sciences | 29 (16.4%) | 17.66 | 17.00 |  |  | 15.21 | 16.00 |  |  |
| Business | 32 (18.1%) | 17.69 | 18.00 |  |  | 14.88 | 14.00 |  |  |
| Education | 35 (19.8%) | 16.66 | 16.00 |  |  | 15.46 | 15.00 |  |  |
| Health Sciences and Human Services | 25 (14.1%) | 17.80 | 18.00 |  |  | 14.72 | 14.00 |  |  |
| Liberal and Fine Arts | 31 (17.5%) | 17.29 | 17.00 |  |  | 14.58 | 14.00 |  |  |
| Science and Technology | 25 (14.1%) | 16.56 | 16.00 |  |  | 14.28 | 14.00 |  |  |
| **Campus Residence** |  |  |  | 3513.50 | 0.83 |  |  | 4086.00 | 0.12 |
| On-campus | 64 (36.2%) | 17.23 | 18.00 |  |  | 14.27 | 14.00 |  |  |
| Off-campus | 112 (63.3%) | 17.26 | 17.00 |  |  | 15.27 | 15.00 |  |  |
| **Health Insurance** |  |  |  | 2681.50 | 0.60 |  |  | 2652.50 | 0.53 |
| Yes | 135 (76.3%) | 17.34 | 7.00 |  |  | 14.96 | 14.00 |  |  |
| No | 42 (23.7%) | 17.02 | 9.00 |  |  | 14.64 | 14.00 |  |  |
| *Statistically significant at p < 0.05 | | | | |  |  |  |  |  |
