# Supplementary material for: Unmasking the impact of COVID-19 on the mental health of college students: a cross-sectional study
Source: Front Psychiatry. 2024 Nov 18;15:1453323. doi: 10.3389/fpsyt.2024.1453323 (PMC11608972; doi:10.3389/fpsyt.2024.1453323)
Supplement: Supplementary file 3 [file Table3.docx]

| **Supplemental Table 3. Demographic and Other Characteristics’ Influences on LD Composite Score: Analysis of Total Sample and Cases with Comorbid Depression and Anxiety** | | | | | | | | |
| --- | --- | --- | --- | --- | --- | --- | --- | --- |
|  | **LD Composite Score** | | | | | | | |
| **Variable** | **All (n = 571)** | | | | **^#^Comorbid (n = 177)** | | | |
|  | **x̄** | **M** | **KW/MW** | **p** | **x̄** | **M** | **KW/MW** | **p** |
| **Gender** |  |  | 12.78 | < 0.01* |  |  | 6.59 | 0.04* |
| Male | 3.49 | 3.00 |  |  | 4.17 | 3.00 |  |  |
| Female | 4.10 | 4.00 |  |  | 5.25 | 5.00 |  |  |
| Other | 6.00 | 6.00 |  |  | 6.43 | 6.00 |  |  |
| **Age** |  |  | 16.58 | 0.01 |  |  | 4.38 | 0.63 |
| 18 | 4.12 | 4.00 |  |  | 6.00 | 6.00 |  |  |
| 19 | 4.26 | 4.00 |  |  | 5.82 | 6.00 |  |  |
| 20 | 4.12 | 4.00 |  |  | 4.66 | 4.00 |  |  |
| 21 | 4.32 | 4.00 |  |  | 4.73 | 4.00 |  |  |
| 22-23 | 4.13 | 4.00 |  |  | 5.06 | 4.00 |  |  |
| 24-31 | 4.45 | 4.00 |  |  | 5.17 | 5.00 |  |  |
| 32+ | 2.90 | 3.00 |  |  | 4.25 | 4.00 |  |  |
| **Race** |  |  | 30.28 | < 0.01* |  |  | 15.50 | < 0.01* |
| Caucasian | 3.66 | 4.00 |  |  | 5.02 | 4.50 |  |  |
| African American | 3.84 | 3.00 |  |  | 3.85 | 3.00 |  |  |
| Hispanic | 5.42 | 5.00 |  |  | 5.97 | 6.00 |  |  |
| Asian | 4.28 | 2.00 |  |  | 8.33 | 7.00 |  |  |
| American Indian | 2.40 | 2.00 |  |  | 1.00 | 1.00 |  |  |
| East Indian | 1.00 | 1.00 |  |  | 0.00 | 0.00 |  |  |
| Other | 3.96 | 4.00 |  |  | 7.00 | 6.00 |  |  |
| **Parent/Guardian’s Education Level** |  |  | 7.03 | 0.32 |  |  | 9.31 | 0.16 |
| Some high school | 4.35 | 4.00 |  |  | 4.33 | 4.00 |  |  |
| High school | 3.74 | 4.00 |  |  | 5.26 | 5.00 |  |  |
| Some college | 3.74 | 4.00 |  |  | 5.03 | 4.00 |  |  |
| Associate’s degree | 4.23 | 4.00 |  |  | 4.31 | 4.00 |  |  |
| Bachelor’s degree | 3.95 | 4.00 |  |  | 5.00 | 5.00 |  |  |
| Master’s degree | 4.40 | 4.00 |  |  | 6.86 | 7.00 |  |  |
| Doctorate degree | 2.54 | 2.00 |  |  | 5.33 | 6.00 |  |  |
| **Non-Traditional Students** |  |  | 35640.00 | 0.11 |  |  | 2594.00 | 0.49 |
| Yes | 3.74 | 3.50 |  |  | 5.40 | 5.00 |  |  |
| No | 4.09 | 4.00 |  |  | 4.96 | 4.00 |  |  |
| **First Generation Students** |  |  | 38837.50 | 0.50 |  |  | 3434.50 | 0.37 |
| Yes | 4.10 | 4.00 |  |  | 5.27 | 5.00 |  |  |
| No | 3.91 | 4.00 |  |  | 4.88 | 4.00 |  |  |
| **Undergraduate Classification** |  |  | 2.80 | 0.42 |  |  | 0.97 | 0.80 |
| Freshman | 3.71 | 3.00 |  |  | 6.09 | 6.00 |  |  |
| Sophomore | 4.15 | 4.00 |  |  | 4.96 | 5.00 |  |  |
| Junior | 4.08 | 4.00 |  |  | 4.96 | 4.00 |  |  |
| Senior | 4.38 | 4.00 |  |  | 4.93 | 4.00 |  |  |
| **Degree Level** |  |  | 5.02 | 0.17 |  |  | 1.39 | 0.50 |
| Undergraduate | 4.10 | 4.00 |  |  | 4.89 | 4.00 |  |  |
| Graduate | 3.64 | 3.00 |  |  | 5.59 | 5.50 |  |  |
| Postgraduate | 4.78 | 4.50 |  |  | 6.33 | 5.00 |  |  |
| Other | 3.33 | 4.00 |  |  | 0.00 | 0.00 |  |  |
| **College** |  |  | 1.71 | 0.89 |  |  | 1.58 | 0.90 |
| Agricultural and Environmental Sciences | 4.04 | 4.00 |  |  | 5.51 | 6.00 |  |  |
| Business | 3.80 | 3.00 |  |  | 5.12 | 4.50 |  |  |
| Education | 4.10 | 4.00 |  |  | 5.05 | 5.00 |  |  |
| Health Sciences and Human Services | 3.96 | 4.00 |  |  | 4.68 | 4.00 |  |  |
| Liberal and Fine Arts | 4.08 | 4.00 |  |  | 5.12 | 4.00 |  |  |
| Science and Technology | 4.13 | 4.00 |  |  | 4.64 | 4.00 |  |  |
| **Campus Residence** |  |  | 37105.00 | 0.29 |  |  | 4353.00 | 0.02* |
| On-campus | 3.81 | 4.00 |  |  | 4.40 | 4.00 |  |  |
| Off-campus | 4.07 | 4.00 |  |  | 5.37 | 5.00 |  |  |
| **Health Insurance** |  |  | 33295.00 | < 0.01* |  |  | 3395.50 | 0.05* |
| Yes | 3.73 | 4.00 |  |  | 4.84 | 4.00 |  |  |
| No | 5.01 | 5.00 |  |  | 5.71 | 6.00 |  |  |
| *Statistically significant at p < 0.05; **^#^**Anxiety and Depression | | | | | | | | |
