# Supplementary material for: Unmasking the impact of COVID-19 on the mental health of college students: a cross-sectional study
Source: Front Psychiatry. 2024 Nov 18;15:1453323. doi: 10.3389/fpsyt.2024.1453323 (PMC11608972; doi:10.3389/fpsyt.2024.1453323)
Supplement: Supplementary file 4 [file Table4.docx]

| **Supplementary Table 4. Association Between Learning Difficulties and Depression/Anxiety: Independent and Combined Analysis (N = 571)** | | | | | | | | | | | | | |
| --- | --- | --- | --- | --- | --- | --- | --- | --- | --- | --- | --- | --- | --- |
|  | **Depression Cases (Yes/No)** | | | | | **Anxiety Cases (Yes/No)** | | | | | **Anxiety and Depression Cases (Yes/No)** | | |
|  | **N** | | **V** | | **P** | **N** | | | **V** | **P** | **N** | **V** | **P** |
| **Overall** |  | | 0.13 | | < 0.01* |  | | | 0.12 | <0.01* |  | 0.13 | < 0.01* |
| Trouble with any learning modality | 208 (92.4%) | |  | |  | 211 (92.1%) | | |  |  | 166 (93.8%) |  |  |
| Did not | 17 (7.6%) | |  | |  | 18 (8.3%) | | |  |  | 11 (6.2%) |  |  |
| **Among those that had trouble** |  | | | | | | | | | | | | |
| Online | 115 (54.0%) | | 0.07 | | 0.12 | 114 (54.0%) | | | 0.04 | 0.30 | 95 (57.2%) | 0.09 | 0.02* |
| Hyflex | 40 (18.8%) | | 0.03 | | 0.44 | 45 (21.3%) | | | 0.08 | 0.08 | 32 (19.3%) | 0.03 | 0.44 |
| Face-to-face | 21 (9.9%) | | 0.02 | | 0.62 | 19 (9.0%) | | | 0.17 | 0.06 | 14 (8.4%) | 0.35 | 0.04* |
| Not sure | 30 (14.1%) | | 0.01 | | 0.91 | 22 (10.4%) | | | 0.09 | 0.04* | 18 (10.8%) | 0.06 | 0.16 |
| Other | 7 (3.3%) | | 0.04 | | 0.29 | 11 (5.2%) | | | 0.02 | 0.56 | 7 (4.2%) | 0.01 | 0.84 |
|  |  | **Composite PHQ-9**  **(Depression) Score** | | | | **Composite GAD-7**  **(Anxiety) Score** | | | | |  |  |  |
|  | **N** | **x̄** | **M** | **MW/KW** | **p** | **x̄** | **M** | **MW/KW** | **p** |  |  |  |  |
| **Overall** |  |  |  | 25253.00 | < 0.01* |  |  | 23,929.00 | < 0.01* |  |  |  |  |
| Trouble with any learning modality^ | 498 (87.22%) | 9.45 | 8.00 |  |  | 7.93 | 7.00 |  |  |  |  |  |  |
| Did not | 73 (12.78%) | 5.15 | 2.00 |  |  | 4.94 | 3.00 |  |  |  |  |  |  |
| **Among those that had trouble** |  |  |  | 4.70 | 0.32 |  |  | 5.64 | 0.23 |  |  |  |  |
| Online | 269 (47.11%) | 9.69 | 9.00 |  |  | 8.11 | 7.00 |  |  |  |  |  |  |
| Hyflex | 93 (16.29%) | 9.82 | 9.00 |  |  | 8.26 | 7.00 |  |  |  |  |  |  |
| Face-to-face | 37 (6.48%) | 9.73 | 8.00 |  |  | 8.68 | 8.00 |  |  |  |  |  |  |
| Not sure | 75 (13.13%) | 8.41 | 7.00 |  |  | 6.57 | 5.00 |  |  |  |  |  |  |
| Other | 24 (4.2%) | 8.12 | 5.50 |  |  | 7.79 | 5.50 |  |  |  |  |  |  |
| **Individual Analysis** |  |  |  |  |  |  |  |  |  |  |  |  |  |
| **Online** |  |  |  | 46229 | < 0.01* |  |  | 45,083.00 | 0.02* |  |  |  |  |
| Yes | 269 (47.11%) | 9.69 | 9.00 |  |  | 8.11 | 7.00 |  |  |  |  |  |  |
| No | 302 (53.89%) | 8.20 | 7.00 |  |  | 7.05 | 6.00 |  |  |  |  |  |  |
| **Hyflex** |  |  |  | 24905.50 | 0.07 |  |  | 24,719.50 | 0.09 |  |  |  |  |
| Yes | 93 (16.29%) | 9.82 | 8.00 |  |  | 8.26 | 7.00 |  |  |  |  |  |  |
| No | 478 (83.71%) | 8.84 | 9.00 |  |  | 7.47 | 6.00 |  |  |  |  |  |  |
| **Face-to-face** |  |  |  | 10374.00 | 0.61 |  |  | 10,689.00 | 0.84 |  |  |  |  |
| Yes | 37 (6.48%) | 9.73 | 8.00 |  |  | 8.68 | 8.00 |  |  |  |  |  |  |
| No | 534 (93.52%) | 8.84 | 8.00 |  |  | 7.47 | 6.00 |  |  |  |  |  |  |
| **Not sure** |  |  |  | 17627.50 | 0.46 |  |  | 16,561.00 | 0.12 |  |  |  |  |
| Yes | 75 (13.13%) | 8.41 | 7.00 |  |  | 6.57 | 5.00 |  |  |  |  |  |  |
| No | 496 (86.87%) | 8.97 | 8.00 |  |  | 7.70 | 7.00 |  |  |  |  |  |  |
| **Other** |  |  |  | 6129.00 | 0.35 |  |  | 658,805.00 | 0.98 |  |  |  |  |
| Yes | 24 (4.2%) | 8.13 | 5.50 |  |  | 7.79 | 5.50 |  |  |  |  |  |  |
| No | 547 (95.8%) | 8.93 | 8.00 |  |  | 7.54 | 6.00 |  |  |  |  |  |  |
| ^Participants responding to one or more learning difficulty | | | | | | | | | | | |  |  |
| *Statistically significant at p < 0.05 | | | | | | | | | |  |  |  |  |
