# Supplementary material for: Unmasking the impact of COVID-19 on the mental health of college students: a cross-sectional study
Source: Front Psychiatry. 2024 Nov 18;15:1453323. doi: 10.3389/fpsyt.2024.1453323 (PMC11608972; doi:10.3389/fpsyt.2024.1453323)
Supplement: Supplementary file 5 [file Table5.docx]

| **Supplementary Table 5. Influence of COVID-19-Related Factors on Depression and Anxiety Severity (n = 571)** | | | | | | | | | |
| --- | --- | --- | --- | --- | --- | --- | --- | --- | --- |
|  |  | **Composite PHQ-9 (Depression) Score** | | | | **Composite GAD-7 (Anxiety) Score** | | | |
|  | **N** | **M** | **x̄** | **KW/MW** | **p** | **M** | **x̄** | **KW/MW** | **p** |
| **Vaccination Status** |  |  |  | 33436.00 | < 0.01* |  |  | 36000.50 | < 0.01* |
| Vaccinated | 290 (51.0%) | 9.00 | 9.84 |  |  | 7.00 | 8.28 |  |  |
| Unvaccinated | 279 (49.0%) | 6.00 | 7.92 |  |  | 6.00 | 6.81 |  |  |
| **Past COVID-19 Positivity (self)** |  |  |  | 29081.00 | 0.84 |  |  | 29072.50 | 0.43 |
| Yes | 135 (23.6%) | 8.00 | 8.99 |  |  | 7.00 | 7.91 |  |  |
| No | 436 (76.9%) | 8.00 | 8.87 |  |  | 6.00 | 7.44 |  |  |
| **Family’s Past COVID-19 Positivity** |  |  |  | 39775.00 | 0.78 |  |  | 37688.50 | 0.04* |
| Yes | 279 (49.2%) | 8.00 | 8.94 |  |  | 7.00 | 8.13 |  |  |
| No | 289 (50.8%) | 8.00 | 8.84 |  |  | 6.00 | 6.99 |  |  |
| **Residence at COVID-19 Diagnosis** |  |  |  | 1.30 | 0.73 |  |  | 2.61 | 0.46 |
| Residence hall | 21 (15.4%) | 9.00 | 9.90 |  |  | 8.00 | 8.62 |  |  |
| Family | 80 (58.8%) | 7.50 | 8.38 |  |  | 6.00 | 7.81 |  |  |
| Off-campus, not with family | 25 (18.4%) | 8.00 | 9.88 |  |  | 7.00 | 8.84 |  |  |
| Other | 10 (7.4%) | 10.50 | 9.80 |  |  | 5.50 | 6.20 |  |  |
| **Experienced Social Stigma Associated With COVID-19 Infection** |  |  |  | 2572.50 | < 0.01* |  |  | 2532.50 | < 0.01* |
| Yes | 57 (29.7%) | 11.00 | 12.02 |  |  | 10.00 | 10.93 |  |  |
| No | 135 (70.3%) | 6.00 | 7.82 |  |  | 5.00 | 6.79 |  |  |
| **Plans To Get COVID-19 Vaccine (if Unvaccinated)** |  |  |  | 4.48 | 0.21 |  |  | 2.96 | 0.40 |
| Yes | 32 (11.6%) | 8.00 | 9.09 |  |  | 7.00 | 7.22 |  |  |
| No | 151 (54.5%) | 5.00 | 7.42 |  |  | 5.00 | 6.59 |  |  |
| Maybe | 52 (18.8%) | 6.50 | 8.73 |  |  | 6.50 | 7.04 |  |  |
| I don’t know | 42 (15.2%) | 5.00 | 7.24 |  |  | 5.00 | 6.62 |  |  |
