# Supplementary material for: Unmasking the impact of COVID-19 on the mental health of college students: a cross-sectional study
Source: Front Psychiatry. 2024 Nov 18;15:1453323. doi: 10.3389/fpsyt.2024.1453323 (PMC11608972; doi:10.3389/fpsyt.2024.1453323)
Supplement: Supplementary file 6 [file Table6.docx]

| **Supplemental Table 6. Association Between Specific COVID-19 Related Factors and LD Composite Score** | | | | | |
| --- | --- | --- | --- | --- | --- |
| **LD Composite Score** | | | | | |
| **COVID Experiences** |  | **M** | **x̄** | **KW/MW** | **p** |
| **Vaccination Status** |  |  |  | 40474.00 | 0.99 |
| Vaccinated | 290 (51.0%) | 4.00 | 3.93 |  |  |
| Unvaccinated | 270 (47.5%) | 4.00 | 4.01 |  |  |
| **Participants’ Past**  **COVID Positivity Status** |  |  |  | 29528.50 | 0.95 |
| Yes | 135 (24.1%) | 3.00 | 4.17 |  |  |
| No | 436 (77.7%) | 3.00 | 3.95 |  |  |
| **Family’s Past**  **COVID Positivity Status** |  |  |  | 34145.00 | < 0.01* |
| Yes | 279 (49.1%) | 4.00 | 4.38 |  |  |
| No | 289 (50.9%) | 3.00 | 3.64 |  |  |
| **Residence During**  **COVID Diagnosis** |  |  |  | 2.17 | 0.54 |
| Residence hall | 21 (16.7%) | 3.00 | 3.62 |  |  |
| Family | 80 (63.5%) | 3.00 | 4.03 |  |  |
| Off-campus, not with family | 25 (19.8%) | 4.00 | 4.44 |  |  |
| **Experienced Social Stigma Associated with COVID-19 infection** |  |  |  | 3246.50 | 0.09 |
| Yes | 57 (29.7%) | 4.00 | 5.04 |  |  |
| No | 135 (70.3%) | 3.00 | 3.95 |  |  |
| **Plans To Get COVID Vaccine**  **(if Unvaccinated)** |  |  |  | 7.95 | 0.047* |
| Yes | 32 (11.6%) | 4.00 | 5.16 |  |  |
| No | 151 (54.5%) | 4.00 | 3.70 |  |  |
| Maybe | 52 (18.8%) | 3.50 | 3.77 |  |  |
| I don’t know | 42 (15.2%) | 4.00 | 4.48 |  |  |
| **Economic Shocks** | | | | | |
| **Loss of food** |  |  |  | 21549.00 | < 0.01* |
| Yes | 59 (10.3%) | 6.00 | 6.32 |  |  |
| No | 512 (89.7%) | 3.00 | 3.73 |  |  |
| **Loss of job** |  |  |  | 41478.00 | < 0.01* |
| Yes | 179 (31.3%) | 4.00 | 4.74 |  |  |
| No | 392 (68.7%) | 3.00 | 3.67 |  |  |
| **Loss of housing** |  |  |  | 10294.50 | < 0.01* |
| Yes | 29 (5.1%) | 6.00 | 5.41 |  |  |
| No | 542 (94.3%) | 3.00 | 3.93 |  |  |
| **Abuse** | | | | | |
| **Overall** |  |  |  | 13,527.50 | < 0.01* |
| Experienced abuse | 38 (6.6%) | 7.00 | 5.84 |  |  |
| Did not | 534 (93.4%) | 3.00 | 3.87 |  |  |
| **Abuse Type** |  |  |  |  |  |
| Verbal | 37 (97.4%) | 7.00 | 5.73 | 13010.50 | < 0.01* |
| Emotional | 7 (18.4%) | 8.00 | 8.00 | 3366.00 | < 0.01* |
| Physical | 2 (5.3%) | 5.00 | 5.00 | 730.50 | 0.49 |
| **Social media** | | | | | |
|  |  |  |  | 23,272.00 | < 0.01* |
| Used social media | 79 (13.8%) | 5.00 | 4.85 |  |  |
| Did not | 492 (86.2%) | 3.00 | 3.86 |  |  |
| **Comorbidity Present at COVID Diagnosis** | | | | | |
|  |  |  |  | 14,236.50 | 0.19 |
| Had comorbidity | 49 (8.6%) | 4.00 | 4.73 |  |  |
| Did not | 522 (91.4%) | 3.00 | 3.93 |  |  |

*Statistically significant at p < 0.05
