# Supplementary material for: Unmasking the impact of COVID-19 on the mental health of college students: a cross-sectional study
Source: Front Psychiatry. 2024 Nov 18;15:1453323. doi: 10.3389/fpsyt.2024.1453323 (PMC11608972; doi:10.3389/fpsyt.2024.1453323)
Supplement: Supplementary file 7 [file Table7.docx]

| **Supplementary Table 7. Effect of Economic Shocks on Depression and Anxiety Severity (N = 571)** | | | | | | | | | |
| --- | --- | --- | --- | --- | --- | --- | --- | --- | --- |
|  |  | **Composite PHQ-9**  **(Depression) Score** | | | | **Composite GAD-7**  **(Anxiety) Score** | | | |
|  | **N** | **M** | **x̄** | **MW** | **p** | **M** | **x̄** | **MW** | **p** |
| **Loss of food** |  |  |  | 19438.00 | < 0.01* |  |  | 20569.00 | < 0.01* |
| Yes | 59 (10.3%) | 14.00 | 12.12 |  |  | 12.00 | 11.24 |  |  |
| No | 512 (89.7%) | 8.00 | 8.53 |  |  | 6.00 | 7.13 |  |  |
| **Loss of job** |  |  |  | 42219.00 | < 0.01* |  |  | 44552.50 | < 0.01* |
| Yes | 179 (31.3%) | 9.00 | 10.63 |  |  | 9.00 | 9.60 |  |  |
| No | 392 (68.7%) | 7.00 | 8.75 |  |  | 6.00 | 6.61 |  |  |
| **Loss of housing** |  |  |  | 9549.00 | 0.05 |  |  | 9859.00 | 0.02* |
| Yes | 29 (5.1%) | 11.00 | 11.66 |  |  | 11.00 | 10.28 |  |  |
| No | 542 (94.2%) | 8.00 | 8.75 |  |  | 6.00 | 7.41 |  |  |
