# Supplementary material for: Unmasking the impact of COVID-19 on the mental health of college students: a cross-sectional study
Source: Front Psychiatry. 2024 Nov 18;15:1453323. doi: 10.3389/fpsyt.2024.1453323 (PMC11608972; doi:10.3389/fpsyt.2024.1453323)
Supplement: Supplementary file 8 [file Table8.docx]

| **Supplementary Table 8. Relationship Between Abuse Types and Anxiety/Depression Cases:  Independent and Combined Analysis (N = 384)** | | | | | | | | | | | | |
| --- | --- | --- | --- | --- | --- | --- | --- | --- | --- | --- | --- | --- |
|  |  | **Depression Cases**  **Yes/No)** | | | | **Anxiety Cases**  **(Yes/No)** | | | | **Depression and Anxiety Cases**  **(Yes/No)** | | |
|  | **N** | | | **V** | **p** | **N** | | **V** | **p** | **N** | **V** | **p** |
| **Overall** |  | | | 0.159 | < 0.01* |  |  | 0.15 | < 0.01* |  | 0.16 | < 0.01* |
| Experienced abuse | 26 (11.6%) | | |  |  | 26 (11.4%) | |  |  | 22 (12.4%) |  |  |
| Did not | 199 (88.4%) | | |  |  | 203 (88.6%) | |  |  | 155 (87.6%) |  |  |
| **Abuse Type** |  | | |  |  |  |  |  |  |  |  |  |
| Verbal | 25 (96.1%) | | | 0.15 | < 0.01* | 25 (96.1%) | | 0.15 | < 0.01* | 21 (95.5%) | 0.15 | < 0.01* |
| Emotional | 4 (15.4%) | | | 0.04 | 0.33 | 3 (11.5%) | | 0.01 | 0.88 | 3 (13.6%) | 0.03 | 0.50 |
| Physical | 1 (3.8%) | | | 0.01 | 0.76 | 1 (3.8%) | | 0.01 | 0.78 | 1 (4.5%) | 0.02 | 0.56 |
|  |  | **Composite PHQ-9**  **(Depression) Score** | | | | **Composite GAD-7**  **(Anxiety) Score** | | | |  |  |  |
|  | **N** | **x̄** | **M** | **MW** | **p** | **x̄** | **M** | **MW** | **p** |  |  |  |
| **Overall** |  |  |  | 8,565.50 | < 0.01* |  |  | 8,591.50 | < 0.01* |  |  |  |
| Experienced abuse | 38 (11.1%) | 14.50 | 15.00 |  |  | 12.11 | 12.50 |  |  |  |  |  |
| Did not | 304 (88.9%) | 8.50 | 7.00 |  |  | 7.23 | 6.00 |  |  |  |  |  |
| **Abuse Type^** |  |  |  |  |  |  |  |  |  |  |  |  |
| Verbal | 37 (97.4%) | 14.19 | 15.00 | 8267.00 | < 0.01* | 11.92 | 12.00 | 8310.00 | < 0.01* |  |  |  |
| Emotional | 7 (18.4%) | 13.00 | 14.00 | 2566.50 | 0.17 | 10.00 | 7.00 | 2459.00 | 0.26 |  |  |  |
| Physical | 2 (5.2%) | 14.00 | 14.00 | 1,540.00 | 0.72 | 8.00 | 8.00 | 1,479.00 | 0.89 |  |  |  |
| ^Participants may have responded to more than one category | | | | | | |  |  |  |  |  |  |
| *Statistically significant at p < 0.05 | | | | | | |  |  |  |  |  |  |
