# Supplementary material for: Unmasking the impact of COVID-19 on the mental health of college students: a cross-sectional study
Source: Front Psychiatry. 2024 Nov 18;15:1453323. doi: 10.3389/fpsyt.2024.1453323 (PMC11608972; doi:10.3389/fpsyt.2024.1453323)
Supplement: Supplementary file 9 [file Table9.docx]

| **Supplementary Table 9. Relationship Between Physical Comorbidity Status and Depression/Anxiety Cases: Independent and Combined Analysis**  **(N = 571)** | | | | | | | | | | | | |
| --- | --- | --- | --- | --- | --- | --- | --- | --- | --- | --- | --- | --- |
|  | **Depression Cases** | | | | | **Anxiety Cases** | | | | **Depression and Anxiety Cases** | | |
|  | **N** | | | **V** | **p** | **N** | | **V** | **p** | **N** | **V** | **p** |
|  |  | | | 0.1 | 0.02* |  | | 0.11 | 0.01* |  | 0.11 | 0.01* |
| Physical Comorbidity | 27 (12.0%) | | |  |  | 28 (12.2%) | |  |  | 23 (13.0%) |  |  |
| No Physical Comorbidity | 198 (88.0%) | | |  |  | 201 (87.8%) | |  |  | 154 (87.0%) |  |  |
|  |  | **Composite PHQ-9**  **(Depression) Score** | | | | **Composite GAD-7**  **(Anxiety) Score** | | | |  |  |  |
|  | **N** | **x̄** | **M** | **MW** | **p** | **x̄** | **M** | **MW** | **p** |  |  |  |
|  |  |  |  | 15,360.5 | 0.02* |  |  | 16,079.50 | < 0.01* |  |  |  |
| Physical Comorbidity | 49 (8.6%) | 11.18 | 10.00 |  |  | 10.16 | 10.00 |  |  |  |  |  |
| No Physical Comorbidity | 522 (91.4%) | 8.68 | 7.50 |  |  | 7.31 | 3.00 |  |  |  |  |  |
| *Statistically significant at p < 0.05 | | | | | | | | | | |  |  |
