# Supplementary material for: Unmasking the impact of COVID-19 on the mental health of college students: a cross-sectional study
Source: Front Psychiatry. 2024 Nov 18;15:1453323. doi: 10.3389/fpsyt.2024.1453323 (PMC11608972; doi:10.3389/fpsyt.2024.1453323)
Supplement: Supplementary file 10 [file Table10.docx]

| **Supplementary Table 10. Relationship Between Information-Seeking Behaviors and Depression/Anxiety Cases: Independent and Combined Analysis** | | | | | | | | | | | |
| --- | --- | --- | --- | --- | --- | --- | --- | --- | --- | --- | --- |
|  | **Depression Cases (Yes/No)** | | | | **Anxiety Cases (Yes/No)** | | | | **Depression and Anxiety Cases(Yes/No)** | | |
|  | **N** | | **V** | **p** | **N** | | **V** | **p** | **N** | **V** | **p** |
| **Information Type** |  | | 0.17 | < 0.01* |  | | 0.15 | < 0.01* |  | 0.17 | < 0.01* |
| Did not use any information outlet | 8 (3.6%) | |  |  | 4 (1.75%) | |  |  | 2 (1.13%) |  |  |
| Only used traditional media^ | 179 (79.6%) | |  |  | 185 (80.79%) | |  |  | 145 (81.92%) |  |  |
| Only used social media | 15 (6.7%) | |  |  | 20 (8.73%) | |  |  | 12 (6.78%) |  |  |
| Used both traditional and social media | 23 (10.2%) | |  |  | 20 (8.73%) | |  |  | 18 (10.17%) |  |  |
|  | **Composite PHQ-9**  **(Depression) Score** | | | | | **Composite GAD-7**  **(Anxiety) Score** | | | |  |  |
|  | **N (%)** | **x̄** | **M** | **KW** | **p** | **x̄** | **M** | **KW** | **p** |  |  |
| **Information Type** |  |  |  | 17.41 | < 0.01* |  |  | 16.69 | < 0.01* |  |  |
| Did not use any information outlet | 26 (4.6%) | 6.27 | 5.00 |  |  | 4.58 | 2 |  |  |  |  |
| Only used traditional media | 455 (79.7%) | 8.81 | 8.00 |  |  | 7.49 | 6 |  |  |  |  |
| Only used social media | 47 (8.2%) | 8.34 | 7.00 |  |  | 8.09 | 7 |  |  |  |  |
| Used both traditional and social media | 32 (5.6%) | 13.19 | 14.00 |  |  | 10.09 | 10 |  |  |  |  |
| ^TV, newspapers, emails, friends, family, public authorities | | | | | | | | | | | |
| *Statistically significant at p < 0.05 | | | | | | | | | | | |
