# Supplementary material for: Unmasking the impact of COVID-19 on the mental health of college students: a cross-sectional study
Source: Front Psychiatry. 2024 Nov 18;15:1453323. doi: 10.3389/fpsyt.2024.1453323 (PMC11608972; doi:10.3389/fpsyt.2024.1453323)
Supplement: Supplementary file 11 [file Table11.docx]

| **Supplemental Table 11. Impact of Coping Strategies During COVID-19 on Anxiety and Depression Severity and LD Composite Scores** | | | | | | | | | | | | | |
| --- | --- | --- | --- | --- | --- | --- | --- | --- | --- | --- | --- | --- | --- |
|  |  | **Composite PHQ-9**  **(Depression Score)** | | | | **Composite GAD-7**  **(Anxiety Score)** | | | | **LD Composite Score** | | | |
|  | **N** | **x̄** | **M** | **MW** | **p** | **x̄** | **M** | **MW** | **p** | **x̄** | **M** | **MW** | **p** |
| **Meditation** |  |  |  | 31,725.00 | < 0.01* |  |  | 30,158.50 | 0.09 |  |  | 56,188.00 | < 0.01* |
| Yes | 122 (21.4%) | 10.22 | 9.00 |  |  | 8.40 | 7.00 |  |  | 4.50 | 4.00 |  |  |
| No | 449 (78.6%) | 8.54 | 7.00 |  |  | 7.32 | 6.00 |  |  | 3.19 | 3.00 |  |  |
| **Connecting socially with family and friends via Zoom or other electronic platforms** |  |  |  | 35,360.00 | 0.98 |  |  | 34,065.50 | 0.47 |  |  | 73,571.00 | < 0.01* |
| Yes | 182 (31.9%) | 8.90 | 7.50 |  |  | 7.36 | 6.00 |  |  | 4.20 | 4.00 |  |  |
| No | 389 (68.2%) | 8.90 | 8.00 |  |  | 7.64 | 7.00 |  |  | 3.16 | 3.00 |  |  |
| **Working out/exercising/running/jogging/hiking** |  |  |  | 37,129.50 | 0.13 |  |  | 37,261.50 | 0.15 |  |  | 91,230.00 | < 0.01* |
| Yes | 248 (43.4%) | 8.37 | 7.00 |  |  | 7.08 | 6.00 |  |  | 4.21 | 4.00 |  |  |
| No | 323 (56.6%) | 9.30 | 8.00 |  |  | 7.92 | 7.00 |  |  | 3.03 | 3.00 |  |  |
| **Participating in social gatherings while maintaining social distancing, wearing masks, etc.** |  |  |  | 32,823.50 | 0.85 |  |  | 32,495.00 | 1.00 |  |  | 64,353.00 | < 0.01* |
| Yes | 157 (27.5%) | 8.92 | 8.00 |  |  | 7.41 | 7.00 |  |  | 4.00 | 4.00 |  |  |
| No | 414 (72.5%) | 8.89 | 8.00 |  |  | 7.60 | 6.00 |  |  | 3.25 | 3.00 |  |  |
| **Participating in social gatherings without maintaining social distancing, wearing masks, etc.** |  |  |  | 31,951.00 | 0.10 |  |  | 31,316.00 | 0.04* |  |  | 71,173.00 | < 0.01* |
| Yes | 178 (31.2%) | 8.16 | 7.00 |  |  | 6.76 | 6.00 |  |  | 4.12 | 4.00 |  |  |
| No | 393 (68.8%) | 9.23 | 8.00 |  |  | 7.91 | 7.00 |  |  | 3.19 | 3.00 |  |  |
| Engaging in a new or existing hobby |  |  |  | 46,721.00 | < 0.01* |  |  | 45,745.50 | < 0.01* |  |  | 100,751.50 | < 0.01* |
| Yes | 301 (52.7%) | 9.65 | 9.00 |  |  | 8.10 | 7.00 |  |  | 4.12 | 4.00 |  |  |
| No | 270 (47.3%) | 8.06 | 6.00 |  |  | 6.94 | 6.00 |  |  | 2.96 | 2.00 |  |  |
| **Other** |  |  |  | 3,303.50 | 0.97 |  |  | 1,225.50 | 0.24 |  |  | 3,148.50 | 0.84 |
| Yes | 6 (1.0%) | 7.33 | 4.50 |  |  | 4.67 | 2.50 |  |  | 3.25 | 3.50 |  |  |
| No | 565 (98.9%) | 8.92 | 8.00 |  |  | 7.58 | 6.00 |  |  | 3.40 | 3.00 |  |  |
| *Statistically significant at p < 0.05 | | | | | | | | | | | | | |
